# Production of galactitol from galactose by the oleaginous yeast *Rhodosporidium toruloides* IFO0880

**DOI:** 10.1186/s13068-019-1586-5

**Published:** 2019-10-18

**Authors:** Sujit Sadashiv Jagtap, Ashwini Ashok Bedekar, Jing-Jing Liu, Yong-Su Jin, Christopher V. Rao

**Affiliations:** 1 Department of Chemical and Biomolecular Engineering, University of Illinois at Urbana-Champaign, 600 S. Mathews Ave., Urbana, IL 61801 USA; 2 Department of Food Science and Nutrition, University of Illinois at Urbana-Champaign, 600 S. Mathews Ave., Urbana, IL 61801 USA; 3 DOE Center for Advanced Bioenergy and Bioproducts Innovation, University of Illinois at Urbana-Champaign, 600 S. Mathews Ave., Urbana, IL 61801 USA

**Keywords:** *Rhodosporidium toruloides*, Galactose, Galactitol, Lipid, Aldose reductase, Metabolite profiling

## Abstract

**Background:**

Sugar alcohols are commonly used as low-calorie sweeteners and can serve as potential building blocks for bio-based chemicals. Previous work has shown that the oleaginous yeast *Rhodosporidium toruloides* IFO0880 can natively produce arabitol from xylose at relatively high titers, suggesting that it may be a useful host for sugar alcohol production. In this work, we explored whether *R. toruloides* can produce additional sugar alcohols.

**Results:**

*Rhodosporidium toruloides* is able to produce galactitol from galactose. During growth in nitrogen-rich medium, *R. toruloides* produced 3.2 ± 0.6 g/L, and 8.4 ± 0.8 g/L galactitol from 20 to 40 g/L galactose, respectively. In addition, *R. toruloides* was able to produce galactitol from galactose at reduced titers during growth in nitrogen-poor medium, which also induces lipid production. These results suggest that *R. toruloides* can potentially be used for the co-production of lipids and galactitol from galactose. We further characterized the mechanism for galactitol production, including identifying and biochemically characterizing the critical aldose reductase. Intracellular metabolite analysis was also performed to further understand galactose metabolism.

**Conclusions:**

*Rhodosporidium toruloides* has traditionally been used for the production of lipids and lipid-based chemicals. Our work demonstrates that *R. toruloides* can also produce galactitol, which can be used to produce polymers with applications in medicine and as a precursor for anti-cancer drugs. Collectively, our results further establish that *R. toruloides* can produce multiple value-added chemicals from a wide range of sugars.

## Background

Sugar alcohols are commonly used as low-calorie, natural sweeteners [1]. They have also been proposed by the Department of Energy as potential building blocks for bio-based chemicals [2]. Sugar alcohols are naturally found in fruits, vegetables, and mushrooms [3]. In addition, they can be produced from sugars using yeast. Examples include arabitol, erythritol, mannitol, ribitol, and xylitol [4–10]. Production of these sugar alcohols by yeast often results, though not always, from redox imbalances associated with growth on different sugars [4, 5]. The ability of yeast to naturally produce these sugar alcohols from simple sugars provides a potentially safer and more sustainable route than chemical ones based on hydrogenation [11]. Indeed, a number of sugar alcohols are industrially produced using yeast fermentations [12–15].

In this work, we investigated sugar alcohol production in *Rhodosporidium toruloides* IFO0880, a red basidiomycete yeast. This yeast has been principally studied in the context of lipid-based chemical production, because it produces lipids at high titers during growth on a wide range of sugars [16–21]. In addition, it has been genetically engineered to produce carotenoids and terpenoids, indicating that *R. toruloides* can produce a wide range of value-added compounds [22–26].

Recently, *R. toruloides* was also found to produce arabitol, a five-carbon sugar alcohol from xylose, suggesting that it may be a promising host for sugar alcohol production [5]. In particular, *R. toruloides* can grow on a wide range of sugars and can be cultured at high cell densities (> 100 g/L dry cell weight) in fermenters [17–19, 21, 27–29]. In these regards, *R. toruloides* potentially enables a flexible production process where sugar alcohols and lipid-based chemicals can be coproduced.

Motivated by the previous arabitol finding, we tested whether *R. toruloides* is able to produce sugar alcohols from arabinose, cellobiose fructose, galactose, glucose, glycerol, mannose, or sucrose. Among the substrates tested, we observed sugar alcohol production only during growth on galactose, where the cells produced galactitol. We further characterized the pathway involved in galactitol production and identified the critical aldol reductase. Galactitol, in addition to being a low-calorie sweetener, has also been used to produce polymers, with potential applications in drug delivery and tissue engineering [30–32]. In addition, dianhydrogalactitol, a product of galactitol, can be used to treat a variety of cancers [33–36]. Collectively, this work suggests that *R. toruloides* can be used to produce this valuable sugar alcohol.

## Results

### *Rhodosporidium toruloides* produce galactitol during growth on galactose in nitrogen-rich medium

**Fig. 1:**
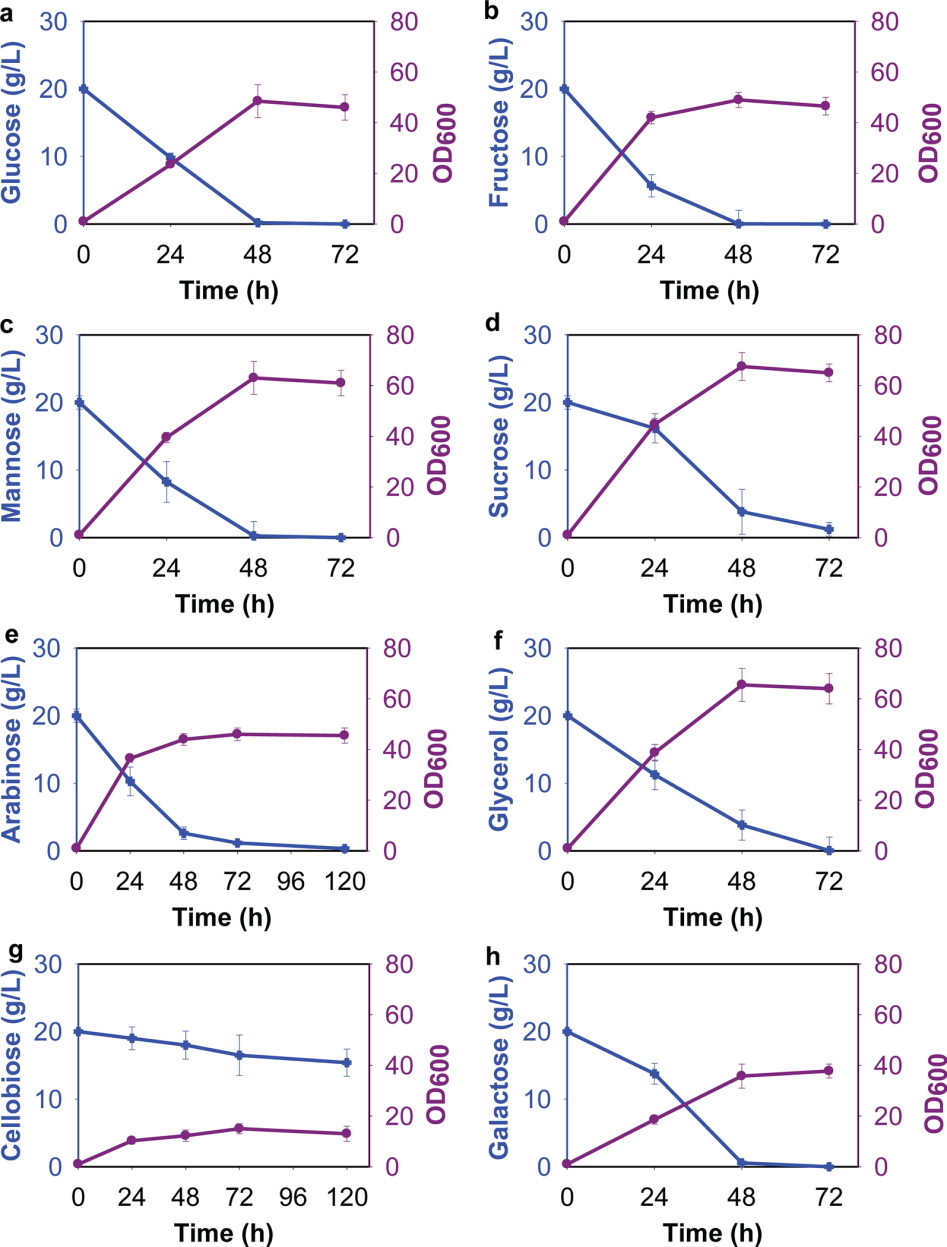
Growth of *R. toruloides* IFO0880 on different sugars at 20 g/L in nitrogen-rich medium: **a** glucose, **b** fructose, **c** mannose, **d** sucrose, **e** arabinose, **f** glycerol, **g** cellobiose, and **h** galactose

To determine whether *R. toruloides* IFO0880 produces any sugar alcohols in addition to arabitol, we measured growth on arabinose, cellobiose, fructose, galactose, glucose, glycerol, mannose, and sucrose in nitrogen-rich medium, the same based medium that induces arabitol production during growth on xylose (Fig. 1). Cells were able to completely consume glucose, fructose, galactose, and mannose after 48 h of shake flask growth. Likewise, cells were able to consume almost 20 g/L sucrose, arabinose, and glycerol after 72 h of shake flask growth. Growth on cellobiose was very slow as compared to the other sugars.

Only during growth on galactose did we observe potential sugar alcohol production as determined by high performance liquid chromatography (HPLC). Analysis of the HPLC chromatogram indicated the presence of an additional metabolite produced by *R. toruloides* (Additional file 1: Figure S1). This metabolite was identified as d-galactitol by gas chromatography–mass spectrometry (GC–MS) and proton nuclear magnetic resonance spectroscopy (^1^H-NMR) (Additional file 2: Figure S2 and Additional file 3: Figure S3).

**Fig. 2:**
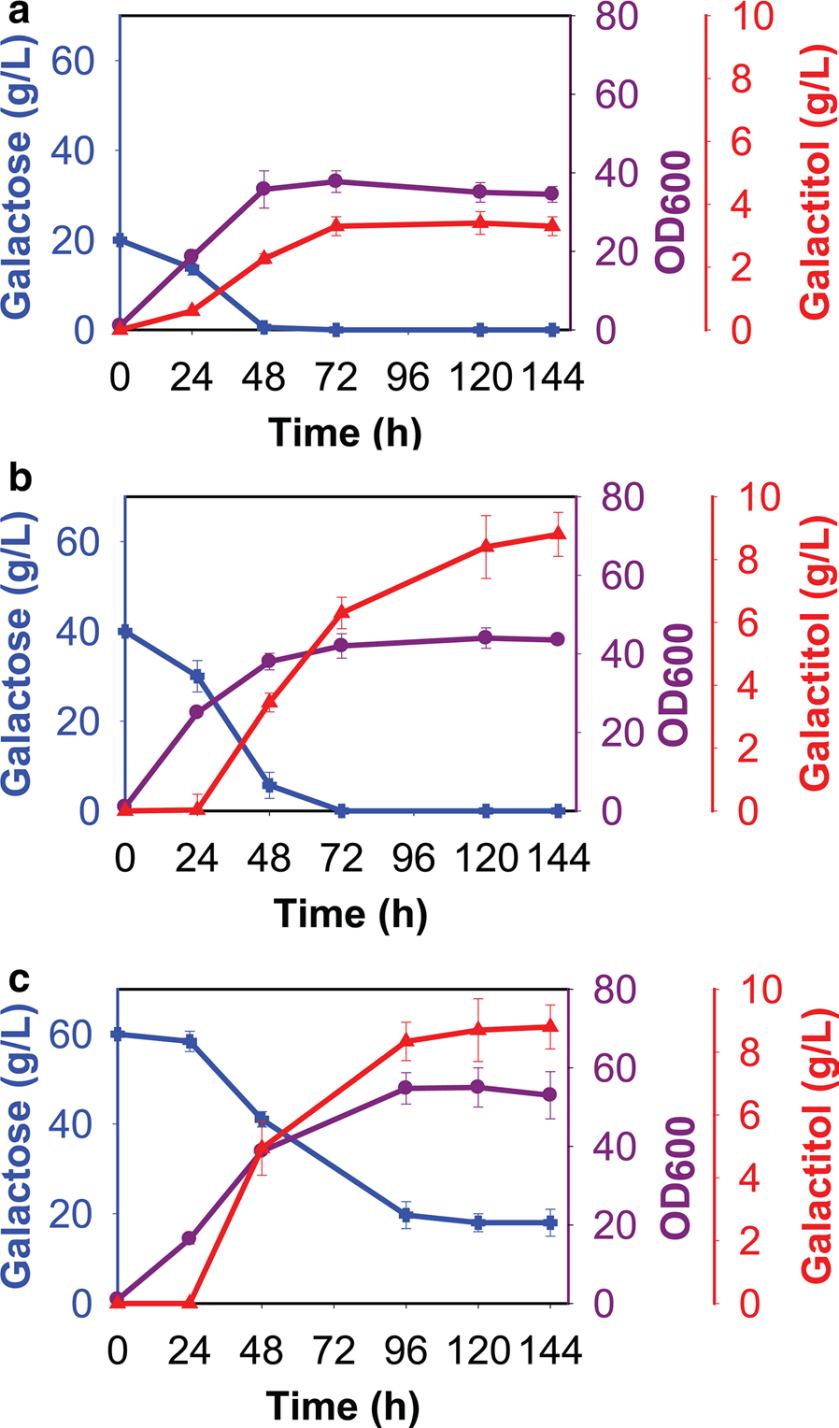
Growth of *R. toruloides* IFO0880 on different concentrations of galactose in nitrogen-rich medium: **a** 20 g/L of galactose, **b** 40 g/L of galactose, **c** 60 g/L of galactose

We next measured the concentration of d-galactitol during growth of *R. toruloides* on galactose in nitrogen-rich medium. d-galactitol was produced at all galactose concentrations tested (Fig. 2). When the initial galactose concentration was 20 or 40 g/L, galactitol production peaked at concentrations of 3.2 ± 0.6 g/L, and 8.4 ± 0.8 g/L, respectively, after 120 h of growth. When the initial galactose concentration was 60 g/L, 8.2 ± 1 g/L of galactitol was produced after 96 h of growth. However, we found that the galactose was not completely consumed when the initial galactose concentration was 60 g/L. This would explain why the galactitol titers were similar during growth on 40 g/L and 60 g/L galactose.

The maximum galactitol production rate and yield were 0.061 g/L/h and 0.22 g/g, respectively (Additional file 11: Table S1). We observed maximal optical densities of 34.5 ± 2, 43.5 ± 2, and 53 ± 6 during growth on 20, 40, and 60 g/L galactose, respectively. We also tested whether *R. toruloides* was able to consume galactitol during growth in nitrogen-rich medium. No consumption was detected (Additional file 4: Figure S4).

### Lipids and galactitol can be produced as co-products

**Fig. 3:**
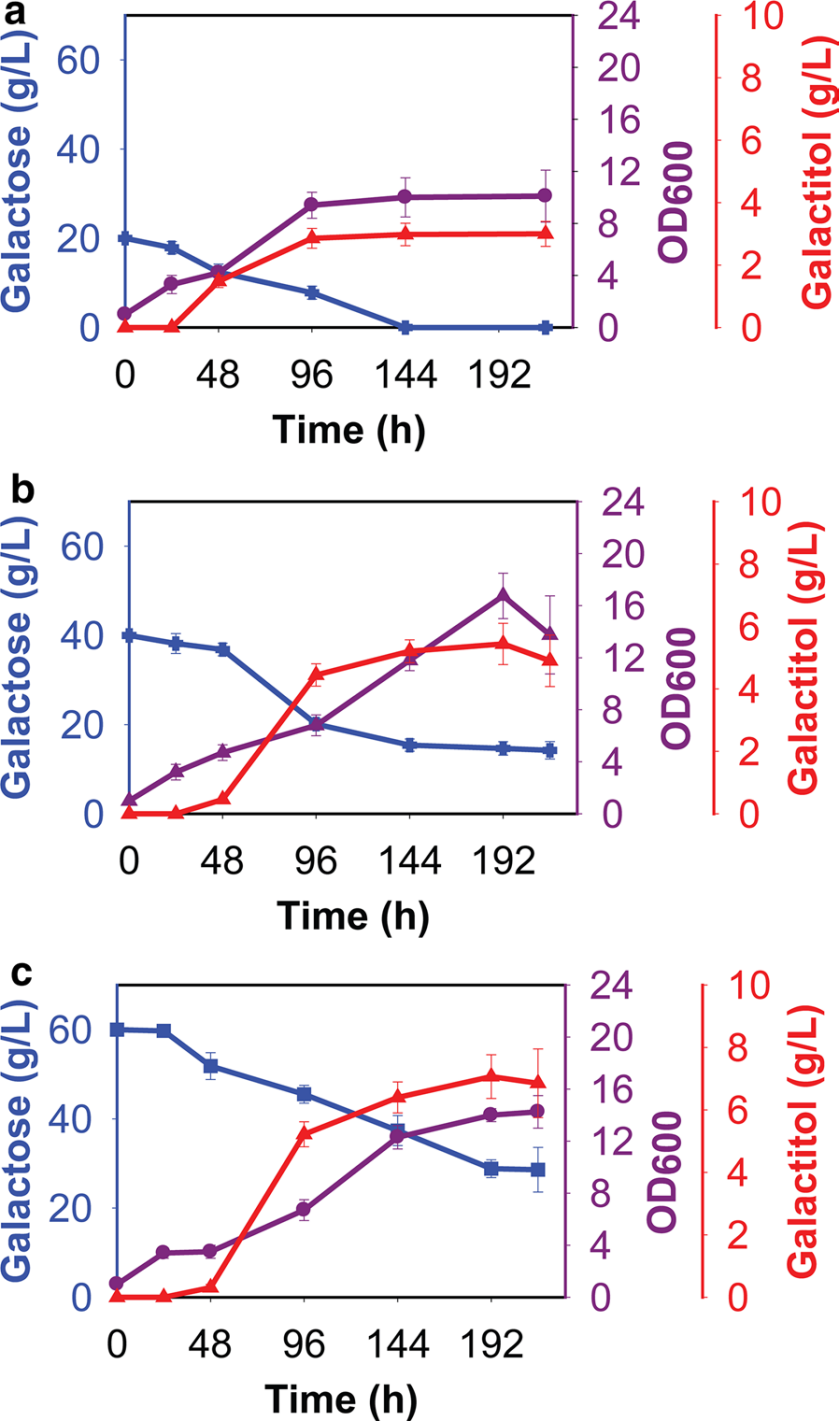
Growth of *R. toruloides* IFO0880 on different concentrations of galactose in nitrogen-poor medium: **a** 20 g/L of galactose, **b** 40 g/L of galactose, **c** 60 g/L of galactose

Oleaginous yeasts are routinely used as hosts for producing lipids from sugars. Lipid production in oleaginous yeast is induced by starving the cells of some essential nutrient, most commonly nitrogen. The results above demonstrate that *R. toruloides* can be used to produce galactitol. They also suggest that *R. toruloides* can be used to simultaneously produce lipids and galactitol, both valuable products. To test this hypothesis, we grew cells in nitrogen-poor medium with 20, 40, or 60 g/L galactose.

d-galactitol was produced during growth in nitrogen-poor medium at the three galactose concentrations tested (Fig. 3). When the initial galactose concentration was 20 g/L, galactitol titers were 3.0 ± 0.3 g/L after 144 h of growth. When the initial galactose concentrations were 40 or 60 g/L, galactitol titers were 5.2 ± 0.5 g/L and 7.4 ± 0.8 g/L, respectively, after 144 h and 192 h of growth. However, we found that galactose was not completely consumed in nitrogen-poor medium when the initial galactose concentration was 40 g/L or 60 g/L. The maximum galactitol production rate and yield were 0.028 g/L/h and 0.136 g/g, respectively, during growth in nitrogen-poor medium (Additional file 11: Table S1).

**Fig. 4:**
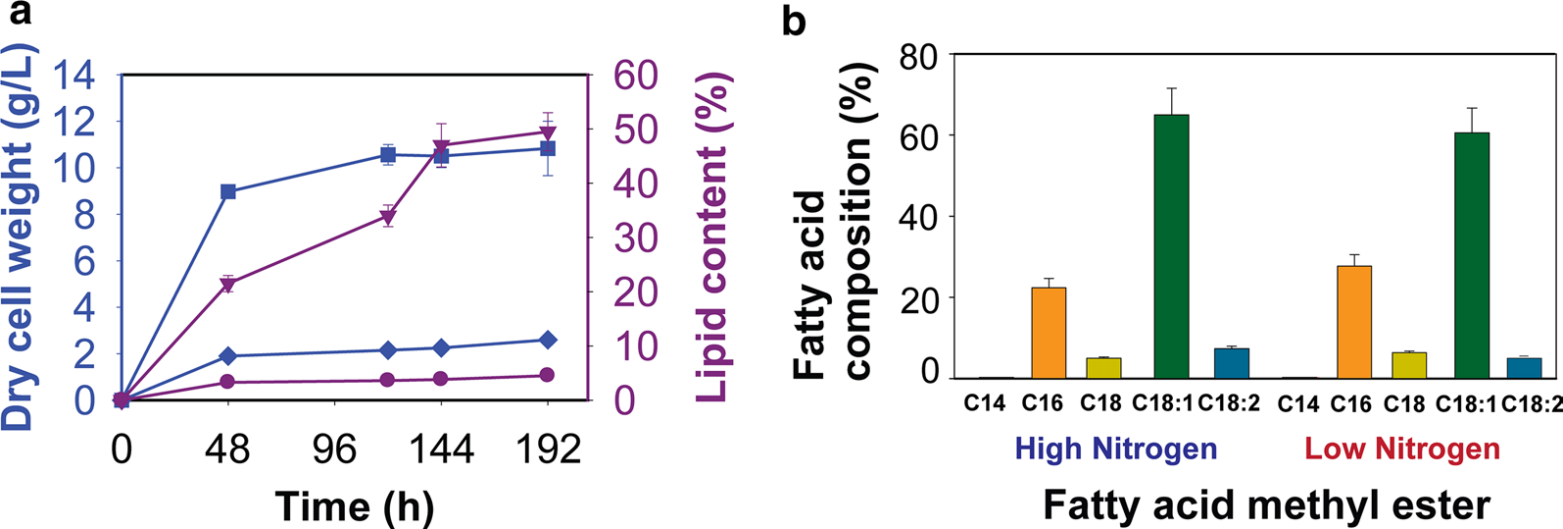
Intracellular lipid and FAME production by *R. toruloides* IFO0880 during growth in nitrogen-poor and nitrogen-rich medium with 40 g/L of galactose: **a** intracellular lipid content and dry cell weight for *R. toruloides* IFO0880. Solid diamonds and solid squares are used to denote dry cell weight of cells grown in nitrogen-poor and nitrogen-rich conditions, respectively. Solid inverted triangles and solid circles are used to denote lipid content of cells grown in nitrogen-poor and nitrogen-rich conditions, respectively. **b** Fatty acid composition as determined by GC–MS. Data show the mean and standard deviation resulting from two biological and two technical replicates

We also analyzed the lipid content and composition in cells grown on 40 g/L galactose in nitrogen-poor and nitrogen-rich medium. In nitrogen-poor medium, the final dry cell weight was 3.1 g/L and lipid content was 45% (Fig. 4a). In nitrogen-rich medium, the final dry cell weight was 10.5 g/L and lipid content was 5%. We also analyzed the composition of the lipid by fatty acid methyl ester analysis using GC–MS. The fatty acids were predominantly oleic (C18:1) and palmitic (C16:0) acid, with some stearic (C18:0) acid during growth on nitrogen-poor and nitrogen-rich medium (Fig. 4b). Similar compositions were observed during growth on glucose and xylose [5, 21].

### *Rhodosporidium toruloides* employs the Leloir pathway for galactose conversion

**Fig. 5:**
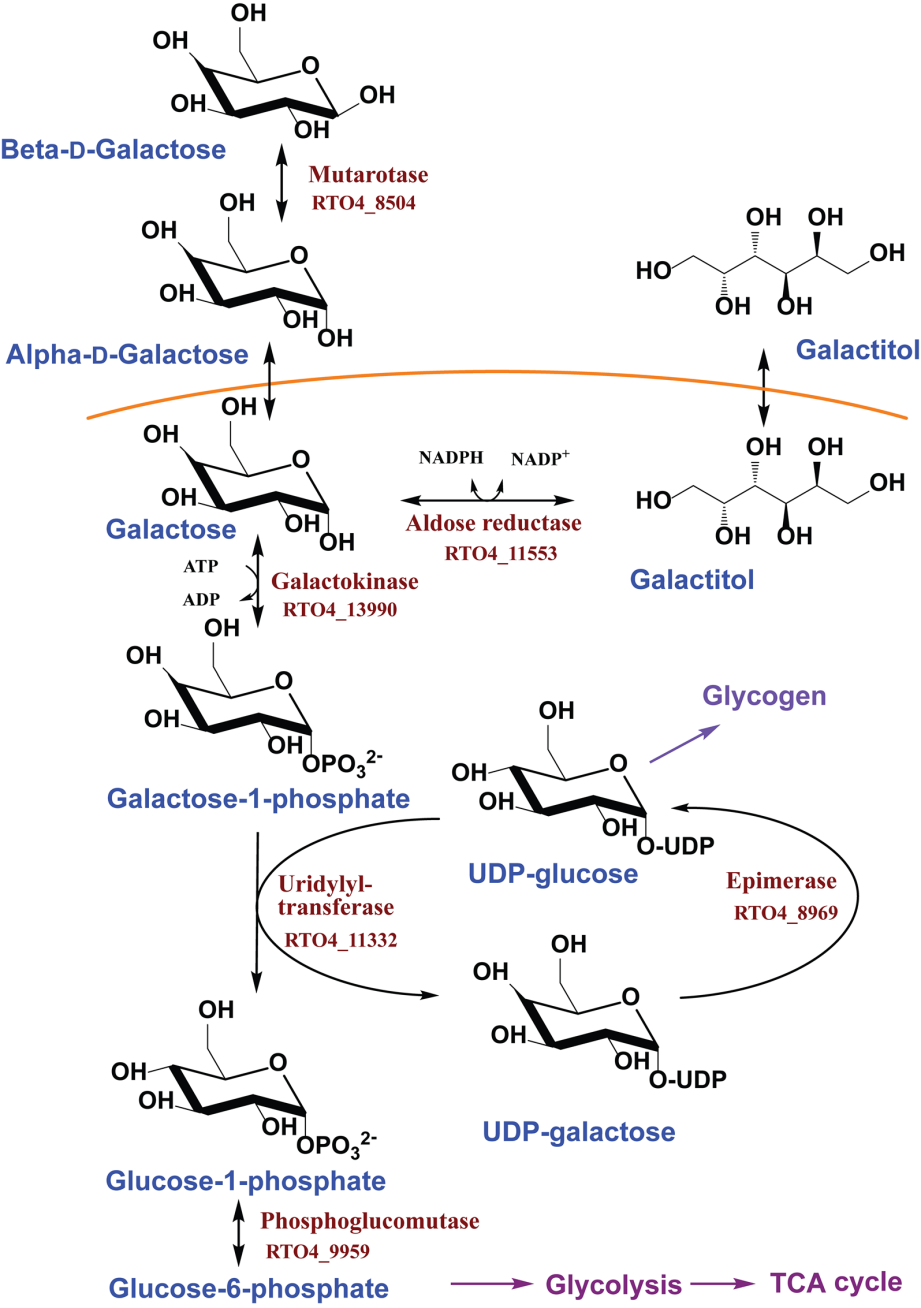
Leloir pathway for galactose metabolism in *R. toruloides* IFO0880

The Leloir pathway is employed for galactose utilization by many yeast, including *Saccharomyces cerevisiae, Kluyveromyces lactis, and Yarrowia lipolytica* [37–40]. *R. toruloides* IFO0880 appears to possess the necessary genes for galactose conversion by the Leloir pathway (Fig. 5). These genes were identified from the draft genome sequence of *R. toruloides* IFO0880 using the annotated genome for *R. toruloides* NP11 as the Refs. [21, 29]. The associated gene identification numbers are provided below next to the associated enzymes.

In the Leloir pathway, β-d-galactose is first converted to α-d-galactose by galactose mutarotase (GM, RTO4_8504). α-d-galactose is then phosphorylated to galactose-1-phosphate by galactokinase (GK, RTO4_13990). It then appears to be converted into galactitol by aldose reductase (AldR, RTO4_11553). In the form of galactose-1-phosphate, the galactose moiety is exchanged with the glucose group from UDP-glucose to generate UDP-galactose, simultaneously releasing glucose-1-phosphate by galactose-1-phosphate uridyl transferase (UT, RTO4_11332). Then, UDP-galactose is epimerized into UDP-glucose by UDP-glucose 4-epimerase (EP, RTO4_8969). In the last step, glucose-1-phosphate is converted to glucose-6-phosphate by phosphoglucomutase (PGM, RTO4_9959). Glucose-6-phosphate then enters into glycolysis.

**Fig. 6:**
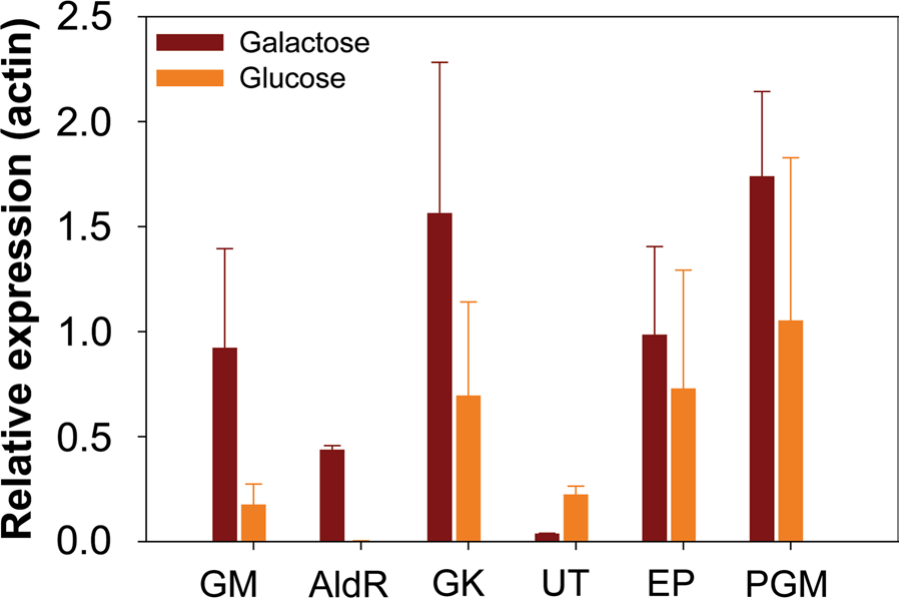
Expression profiles for galactose metabolic genes in *R. toruloides* IFO0880. Gene expression levels were normalized based on the expression of the actin gene. *GM* galactose mutarotase, *AldR* aldose reductase, *GK* galactokinase, *UT* galactose-1-phosphate uridyl transferase, *EP* UDP-glucose 4-epimerase, *PGM* glucose-6-phosphate by phosphoglucomutase

We examined the expression of the genes in the Leloir pathway using quantitative PCR during growth in nitrogen-rich medium containing 2% glucose or 2% galactose (Fig. 6 and Additional file 11: Table S2). All the genes were expressed during growth on galactose. Many were also expressed during growth on glucose, though the relative expression level was often lower. This would suggest that galactose gene expression is subject to glucose catabolite repression, similar to what is observed in *S. cerevisiae*  [41, 42]. However, the relative decrease in expression is not statistically significant due to variable levels of expression. Only in the case of aldose reductase (*AldR*) did we observe strong repression, as we were unable to detect expression during growth on glucose. In addition, we found that galactose-1-phosphate uridyl transferase (*UT*) expression was higher during growth on glucose than on galactose, a result we cannot explain.

### Characterization of galactose reductase from *R. toruloides*

According to the proposed pathway (Fig. 5), an aldose reductase, AdhR, is directly involved in producing galactitol. We expressed and purified a codon-optimized version of the enzyme in *E. coli* (Additional file 11: Table S3). *AldR* gene encodes a polypeptide 290 amino acids in length, with a calculated molecular mass of 32.4 kDa. The molecular weight of the recombinant enzyme was approximately 32 kDa, as determined by SDS-PAGE (Additional file 5: Figure S5). The recombinant enzyme exhibited activity against galactose with NADPH as the coenzyme; no activity was detected with NADH. Thus, AldR is an NADPH-dependent enzyme, like other galactose reductases [43–45]. Next, we determined the optimal pH and temperature for enzyme activity. These were found to be pH 7 and 25 °C (Additional file 6: Figure S6).

**Fig. 7:**
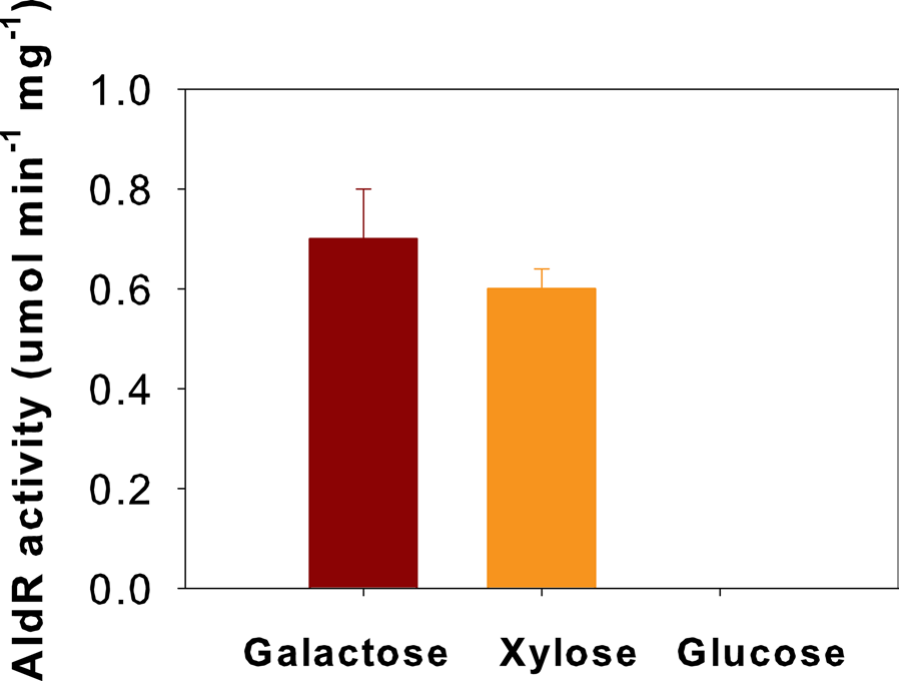
Substrate specificity of aldose reductase (AldR) from *R. toruloides* IFO0880

We next tested the relative activity of AldR on galactose, glucose, and xylose. AldR showed activity on galactose and xylose in the presence of NADPH as the coenzyme (Fig. 7). Among the tested substrates, AldR showed the highest activity on galactose. However, it also exhibited activity on xylose. AldR showed no activity on glucose. Recombinant AldR was then employed to produce galactitol from galactose. Authentic galactose and galactitol were used as controls, and the AldR reaction product was analyzed using HPLC. The product had the same retention time as galactitol (Additional file 7: Figure S7). In the control experiment without AldR enzyme, we did not observe galactitol production or galactose consumption. Collectively, these results suggest that AldR is directly involved in galactitol production.

### Analysis of intracellular metabolites

**Fig. 8:**
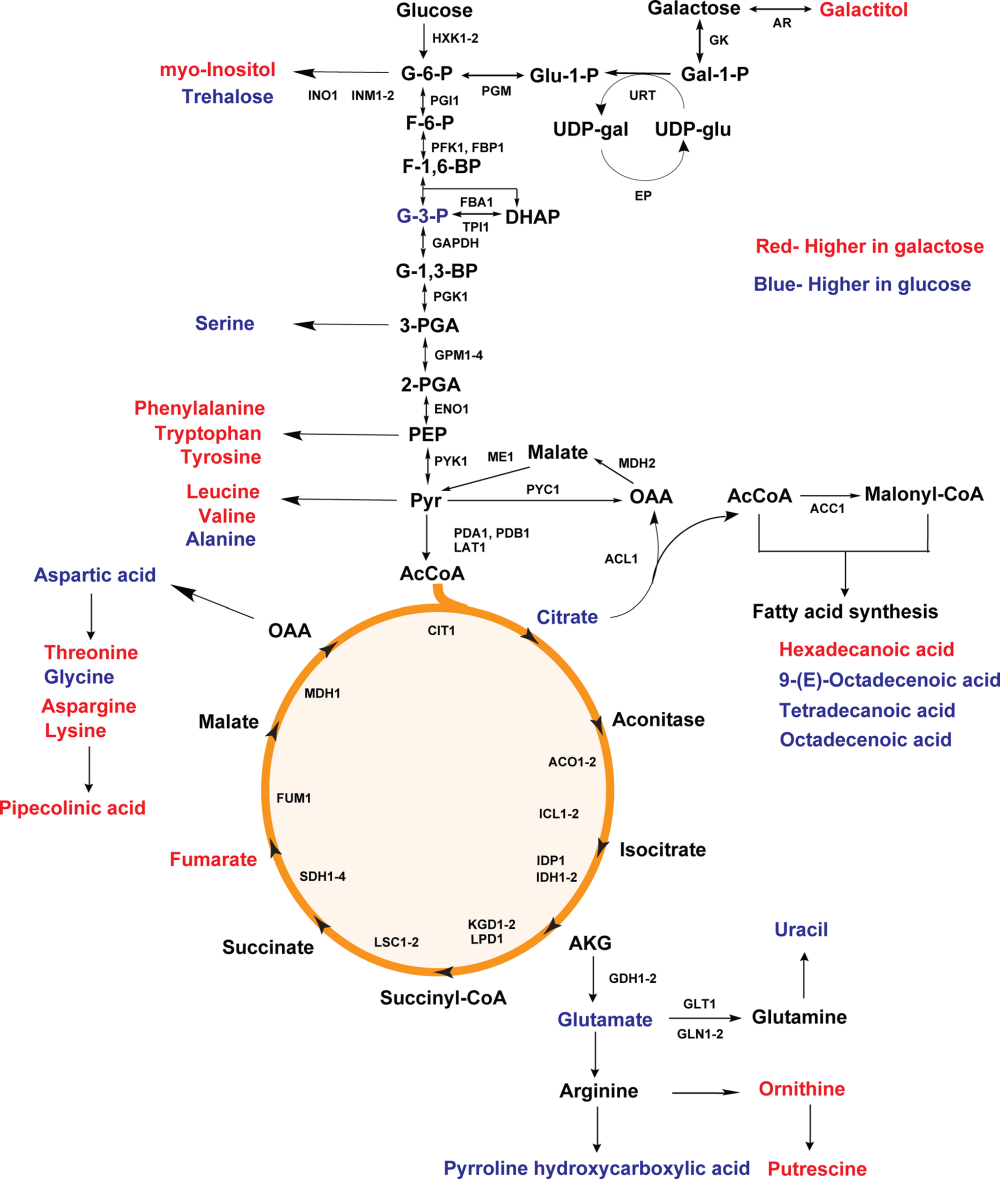
Changes in metabolite concentrations during growth of *R. toruloides* IFO0880 on galactose (GAL) versus glucose (GLC). Metabolites with higher concentration during growth on galactose as shown in red and metabolites with higher concentrations during growth on glucose are shown in blue. *Gal-1-P* galactose-1-phosphate, *UDP-glu* UDP-glucose, *UDP-gal* UDP-galactose, *Glu-1-P* glucose-1-phosphate, *G-6-P* glucose 6-phosphate, *F-6-P* fructose 6-phosphate, *F-1,6-BP* fructose 1,6-bisphosphate, *G-3-P* glyceraldehyde 3-phosphate, *DHAP* dihydroxyacetone phosphate, *G-1,3-BP* glycerate 1,3-diphosphate, *3-PGA* 3-phospho-d-glycerate, *2-PGA* 2-phospho-d-glycerate, *PEP* phosphoenolpyruvate, *Pyr* pyruvate, *AcCoA* acetyl CoA, *AKG* alpha-ketoglutarate, *OAA* oxaloacetate

As far less is known about the growth of *R. toruloides* on galactose than on glucose, we compared the relative concentrations of 50 intracellular metabolites using GC–MS during exponential growth. Among the 50 metabolites analyzed, differences in the concentrations of 29 metabolites were observed during growth on galactose versus glucose (Fig. 8, Additional file 8: Figure S8, Additional file 11: Tables S4, S5).

We observed intracellular galactitol production when *R. toruloides* was grown on galactose. No intracellular galactitol production was observed during growth on glucose. (Additional file 9: Figure S9). We also observed decreased concentrations of the storage metabolite trehalose (0.5×), TCA intermediate citric acid (0.7×), and glycolytic intermediate glycerol 3-phosphate (0.8×) as compared to growth on glucose (Additional file 10: Figure S10). This is consistent with lower metabolic fluxes associated with galactose utilization. Aside from these more obvious changes, we also observed changes in many additional metabolites that elude simple explanations. What these results do show is that galactose utilization induces many changes to central metabolism as compared to glucose metabolism.

## Discussion

*Rhodosporidium toruloides* IFO0880 is capable of growing on multiple sugars. Previous work found that *R. toruloides* produces d-arabitol during growth on xylose in nitrogen-rich medium [5]. In this study, we investigated whether *R. toruloides* produces any additional sugar alcohols. The key finding of the present study is that *R. toruloides* also produces galactitol at relatively high titers (> 8 g/L) during growth on galactose. As galactitol is a valuable product in its own right, these results indicate that *R. toruloides* can be used not only to produce lipid-based chemicals but also this sugar alcohol as well.

*Rhodosporidium toruloides* produces galactitol in both nitrogen-rich and nitrogen-poor medium. In the previous d-arabitol study, arabitol was produced only during growth in nitrogen-rich medium [5]. During growth in nitrogen-poor medium, arabitol was not produced. Likely, this is because xylose was consumed very slowly in nitrogen-poor medium. These observations are consistent with d-arabitol being an overflow metabolite associated with redox imbalances in the cell arising from high fluxes through the xylose metabolic pathway during growth in nitrogen-rich medium [4]. In the case of galactose metabolism, galactitol is also likely an overflow metabolite as more is produced when galactose utilization rates are high, for example during growth in nitrogen-rich medium. However, galactitol production does not appear to be associated with any redox imbalances within the cell. More likely, it is produced to prevent the intracellular accumulation of galactose-1-phosphate, a phosphorylated intermediate of the Leloir pathway (Fig. 5), which is toxic to the cell [44, 46]. The efficient utilization of galactose is limited by conversion of galactose-1-phosphate. Low expression of galactose-1-phosphate uridylyltransferase likely causes a buildup of galactose-1-phosphate in the cell. One way to relieve this buildup would be to produce galactitol [47]. Such a mechanism would explain why *R. toruloides* produces galactitol.

Galactitol has been produced using chemical routes based on hydrogenation [11]. It has also been reported that is produced by several yeast during growth on galactose [48, 49]. In one notable study, thirteen yeasts were screened for galactitol production using galactose as a sole carbon source [49]. Six were found to produce galactitol. The highest titers were obtained with *Candida polymorpha*, which produced 18.7 g/L galactitol from 110 g/L galactose. These titers are greater than those obtained with *R. toruloides*. However, the key advantage of using *R. toruloides* is that it can also co-produce other value-added chemicals, in particular lipids and lipid-based chemicals. In addition to yeast, the bacterium *Mycobacterium smegmatis* was found to produce 7 g/L galactitol from 10 g/L galactose and 10 g/L glucose [48]. This appears to be the only reported case of galactitol production in bacteria.

In addition to native production, *S. cerevisiae* has also been engineered to produce galactitol. In one study, the aldose reductase in *S. cerevisiae* was overexpressed. The resulting strain produced 9 mg/L of galactitol from 5 g/L galactose [50]. In another study, *S. cerevisiae* strain EJ4 was found to produce 0.40 g/L galactitol from 20 g/L galactose after 33 h of growth due to the promiscuous activities of heterologously expressed xylose reductase (XR) and xylitol dehydrogenase (XDH) [51]. This strain was also engineered to produce galactitol from lactose by deleting galactose kinase and heterologously expressing galactitol dehydrogenase [52]. It produced 3.5 g/L galactitol from 40 g/L of lactose, suggesting that lactose may also be a promising substrate for galactitol production.

## Conclusions

*Rhodosporidium toruloides* has traditionally been viewed as a production host for lipids and lipid-based chemicals. Our work demonstrates that this oleaginous yeast can also produce galactitol at high titers during growth on galactose. These results suggest that *R. toruloides* potentially enables a flexible production process that can account for different market conditions by producing a wide range of value-added chemicals from simple sugars.

## Methods

### Strains, media, and growth conditions

*Rhodosporidium toruloides* IFO0880 was obtained from the NITE Biological Resource Center, Japan (NBRC 0880). YPD medium (10 g/L yeast extract, 20 g/L peptone, and 20 g/L glucose) was used for routine growth of *R. toruloides*. The two different media were tested for galactitol production by *R. toruloides*: nitrogen-rich medium (10 g/L yeast extract and 20 g/L peptone) and nitrogen-poor medium [0.75 g/L yeast extract, 1.7 g/L yeast nitrogen base without amino acids and ammonium sulfate, 0.1 g/L (NH_4_)_2_SO_4_, and pH 5.6]. Stationary phase seed cultures were obtained by inoculating single colonies from YPD agar plate into 20 mL YPD liquid medium in 125 mL glass baffled shake flasks. The seed cultures were then used to inoculate 50 mL cultures in 250 mL glass baffled shake flasks with a starting OD_600_ of 1. The cells were then grown at 30 °C and 250 rpm.

### Determination of sugar and polyol concentrations

Sugar and galactitol concentrations were measured using a Shimadzu high-performance liquid chromatography (HPLC) system equipped with an RID-10A refractive index detector, an Aminex HPX-87H carbohydrate analysis column (Bio-Rad Laboratories), and a cation H micro-guard cartridge (Bio-Rad Laboratories). The column and guard cartridge were kept at 65 °C, and 5 mM H_2_SO_4_ was used as a mobile phase at a constant flow rate of 0.6 mL/min. Prior to analysis, culture samples were first pelleted and then the supernatant was passed through 0.22 μm polyethersulfone syringe filter. Peaks were identified and quantified by retention time comparison to authentic standards.

Galactose and galactitol peaks were also measured and confirmed by gas chromatography–mass spectrometry (GC–MS). 200 μL of sample was dried under vacuum and derivatized with 50 μL of methoxyamine hydrochloride (40 mg/mL in pyridine) for 60 min at 50 °C, then with 150 μL of *N*-methyl-*N*-(trimethylsilyl)trifluoroacetamide plus 1% of trimethylchlorosilane at 70 °C for 2 h, and following 2 h incubation at room temperature. Chromatograms were acquired using a GC–MS system (Agilent) consisting of an Agilent 7890 gas chromatograph, an Agilent 5975 MSD and an HP 7683B autosampler. Gas chromatography was performed on a ZB-5MS (60 m × 0.32 mm I.D. and 0.25 mm film thickness) capillary column (Phenomenex). Inlet and MS interface temperatures were 230 °C, and the ion source temperature was adjusted to 230 °C. An aliquot of 1 mL was injected with a split ratio of 40:1. Helium carrier gas was kept at a constant flow rate of 2.4 mL/min. The temperature program was: 5 min isothermal heating at 70 °C, followed by an oven temperature increase of 5 °C per min to 310 °C and a final 10 min at 310 °C. The mass spectrometer was operated in a positive electron impact mode at 69.9 eV ionization energy at m/z 50–800 scan range. The spectra of all chromatogram peaks were evaluated using the AMDIS 2.71 (NIST) and authentic standards.

Galactitol peak was also confirmed by proton nuclear magnetic resonance (^1^H-NMR) spectroscopy. Cells were grown for 72 h on 40 g/L galactose in nitrogen-rich media and centrifuged at 16,000×*g* for 10 min. 600 μL of supernatant was mixed with 10% deuterium oxide. All 1D proton spectra were collected at 25 °C on an Agilent 750 MHz VNMRS spectrometer equipped with a 5 mm triple-resonance indirect detection probe with Z PFG gradient capability [11]. The standard 1D presaturation pulse sequence was used, and water suppression was achieved with a low CW power irradiated at water resonance for 1.5 s. Each spectrum was collected with a 90° pulse angle of 10.5 ms, 32 scans and 2 s relaxation delay between scans. A solvent peak (H_2_O) was used as the chemical shift reference. Mnova 14.0.1 (Mestrelab Research) was used for spectral processing and analysis. Each spectrum was processed with 0.6 Hz line broadening, 4 times zero-filling, auto-phase and auto-baseline correction.

### Lipid and dry cell weight measurements

Lipids were also measured using the modified sulpho-phospho-vanillin lipid assay [30, 53, 54]. The appropriate yeast suspension was centrifuged and then the cell pellet washed twice using sterile water. The washed cell pellet was mixed with 1 mL of 18 M sulfuric acid in a glass test tube and heated at 100 °C for 10 min in a dry heating bath. The reaction was cooled for 20 min in an ambient water bath. 2.5 mL of freshly prepared vanillin–phosphoric acid was added and reacted for 15 min at 37 °C. The test tube was cooled for 15 min in a water bath at ambient temperature. The absorbance of each reaction was measured at 530 nm against a reference sample prepared in water using the Tecan Infinite M1000 Pro microplate reader. Absorbance measurements were converted to lipid concentration using a calibration curve prepared using refined corn oil. Corn oil (100 mg) was dissolved in 2:1 chloroform:methanol (20 mL) and a stock solution was loaded into an assay mixture at 50–250 µg. A standard curve was run with each set. Vanillin–phosphoric acid solution was prepared freshly by dissolving 0.12 g vanillin in 20 mL dH_2_O, and adjusting the volume to 100 mL with 85% *o*-phosphoric acid. This assay was validated previously for various oleaginous yeast lipids, and common media ingredients were found to provide minimal interference [30, 54].

Cell growth was measured by the optical absorbance at 600 nm (OD_600_). Dry cell weights (DCW) were determined as follows. Culture samples (1 mL) were collected into pre-weighed tubes and centrifuged at 16,000×*g* for 5 min. Supernatant was discarded. Pellets were then washed twice with 50 mM phosphate buffered saline. Washed pellets were dried to constant weight at 65 °C for 24 to 48 h. The tubes were then weighed.

### Measurement of fatty acid composition

Fatty acid compositions were determined by GC–MS. The fatty acids were first derivatized using the method of Lepage and Roy [55]. Briefly, lyophilized samples (1 mL) were first resuspended in 2 mL of a 20:1 mixture of methanol and acetyl chloride solution and 2 mL of hexane. 25 mg/mL tridecanoic acid dissolved in a 3:2 methanol:benzene mixture was then added (2.5 μL) as an internal standard. The mixture was then incubated in a dry bath at 100 °C for 30 min in a sealed glass tube with a screw cap. After cooling down in room temperature, 1 mL water was added to induce phase separation. This process generates fatty acid methyl esters from all the lipid compounds. The upper organic phase containing the fatty acid methyl esters was collected for analysis by GC/MS. Samples (2 μL) were injected at a 0:1 split ratio using hexane as a solvent. Helium carrier gas was used at a pressure of 121.7 kPa and a flow rate of 1.0 mL/min. The injection port temperature was set at 250 °C. Column temperature started at 30 °C and increased to 250 °C at a rate of 10 °C/min. Eluent from the GC entered an ionization chamber at 250 °C and was measured at a full scan between 15 and 250 amu. Species identity was verified by comparison of mass spectra to the analytical standards in the Sigma-Aldrich’s FAME mix (C8–C24) and NIST mass spectral library. Fatty acid methyl esters were quantified by peak area. Four replicates were analyzed for each sample (obtained from two biological replicates and two technical replicates).

### Quantitative PCR

Cells were harvested after 36 h of shake flask growth in YP medium containing glucose or galactose at 30 °C and then mechanically disrupted using acid-washed glass beads (425–600 μm) six times in a FastPrep-24 homogenizer with 6 m/s beating for 20 s. Total mRNA was then extracted using Qiagen’s RNeasy Mini kit. Total mRNA (2 µg) was then treated with TURBO DNA-free DNase using the TURBO DNA-free kit (Ambion) to remove genomic DNA. cDNA was synthesized from mRNA using the Bio-Rad’s iScript cDNA synthesis kit. The qPCR experiments were carried out using a Roche LightCycler 480 system with the SsoAdvanced Universal SYBR Green Supermix kit (Bio-Rad). Primers were designed using the online PrimerQuest tool provided by Integrated DNA Technologies and are listed in Additional file 11: Table S2. Endogenous actin gene (*ACT*, RHTO_03560) was used as an internal control. All data points were collected from three biological replicates.

### Aldose reductase cloning and characterization

Cloning and protein expression were performed in *Escherichia coli* strains DH5 and BL21(DE3), respectively. *E. coli* was grown at 37 °C in Luria–Bertani (LB) medium (15 g/L yeast extract, 10 g/L tryptone, and 10 g/L NaCl). Kanamycin was used at a concentration of 40 μg/mL.

AldR was expressed from a T7 promoter using the plasmid pET-28(a). Primers used for this study are listed in Additional file 11: Table S2. *R. toruloides* IFO0880 genomic sequence (GenBank: LCTV02000005.1) was accessed from National Center for Biotechnology Information (http://www.ncbi.nlm.nih.gov), and the aldose reductase sequence (Protein Id: 11553 and transcript Id: 11681) was accessed from the JGI Genome Portal. Aldose reductase sequence was codon optimized by Integrated DNA Technologies for expression in *E. coli* (GenBank accession number: MN057734; Additional file 11: Table S3). The overexpression vector was constructed first by PCR amplifying the aldose reductase gene *aldR* and then cloning the fragment into pET-28(a) using the restriction enzymes *Bam*HI-HF and *Hind*III-HF with an N-terminal His_6_-tag.

*Escherichia coli* BL21 (DE3) containing the *aldR* gene was grown overnight at 37 °C on a rotary shaker at 250 rpm. The culture was then sub-cultured 1:100 into fresh LB medium containing kanamycin and grown at 37 °C with shaking at 250 rpm until the optical density at 600 nm reached 0.6–0.8. Aldose reductase expression was induced by adding isopropyl-β-d-1-thiogalactopyranoside to a final concentration of 0.5 mM. Cells were grown at 25 °C for additional 16 h at 200 rpm. Cell lysate was sonicated six times using 40% amplitude for 20 s on ice. AldR lysate was clarified by centrifugation at 13,000×*g* for 20 min and then passed through a 0.45 μm filter. AldR was purified by loading the lysate onto three 5 mL HiTrap Chelating HP columns charged with 0.1 M NiSO_4_ and installed on an AKTA prime FPLC system (GE Healthcare). The cell-free extract was applied onto a column previously equilibrated with binding buffer (50 mM NaH_2_PO_4_, 300 mM NaCl, 10 mM imidazole, and pH 8.0). The unbound proteins were removed from the column with a washing buffer (50 mM NaH_2_PO_4_, 300 mM NaCl, 30 mM imidazole and pH 8.0). AldR was eluted from the column with an elution buffer (50 mM NaH_2_PO_4_, 300 mM NaCl, 250 mM imidazole, and pH 8.0). Enzyme fractions were analyzed by 10–12% SDS-PAGE (Bio-Rad) and visualized by staining with SimplyBlue SafeStain (Invitrogen). Protein concentrations were determined using Coomassie Plus (Bradford) assay kit (Pierce). Molecular weight of the protein was determined by SDS-PAGE analysis.

Aldose reductase activity was determined spectrophotometrically (Cary 100 Bio UV–Visible Spectrophotometer, Agilent) by monitoring the rate of NADP^+^ (*ε* = 5.62 mM^−1^ cm^−1^) or NADPH (*ε* = 5.12 mM^−1^ cm^−1^) formation at 340 nm. The standard reaction mixture (1 mL) contained 20 mM Tris–HCl buffer (pH 7.0), 10 mM of substrate and an appropriate amount (100–1000 µg) of the enzyme. The reaction was started by adding NAPDPH or NADP^+^ to the mixture. One unit of aldose reductase activity was defined as the amount of enzyme that catalyzes the formation of 1 µmol NADP^+^ or NAPDPH per minute. The reaction mixture consisted of 1 mL 20 mM Tris–HCl buffer with pH 7.5, 10 mM NADPH, 10 mM galactose, and purified AldR enzyme. After reacting for overnight at 25 °C, the mixture of products was centrifuged at 12,000×*g* for 20 min and the supernatant was analyzed by HPLC.

### Analysis of intracellular metabolites

For intracellular metabolite analysis, a fast filtration sampling method was used as previously described with a slight modification [56]. Briefly, 0.5 mL of culture grown until the exponential phase was collected and vacuum filtered using a vacuum manifold system (Vac-Man^®^ Laboratory Vacuum Manifold, Promega) assembled with a nylon membrane filter (0.45 µm pore size, 13 mm diameter, Whatman, Piscataway, NJ, USA) and a filter holder (Millipore). The filtered cell culture was then washed with 2.5 mL of distilled water at room temperature. The entire process for fast filtration was finished within 1 min. The filter membrane containing the washed cells was quickly mixed with 1 mL of a pre-chilled acetonitrile/water mixture (1:1, v/v) and 100 µL of glass beads. The extraction mixture was vortexed for 3 min to extract intracellular metabolites of *R. toruloides* IFO0880 by disruption of the cell membrane. The extraction mixture was then centrifuged at 16,000×*g* for 3 min at 4 °C. 0.8 mL of supernatant containing the intracellular metabolites was dried using a speed vacuum concentrator for 6 h.

Prior to GC/MS analysis, intracellular metabolites were derivatized by methoxyamination and trimethylsilylation. For methoxyamination, 10 µL of 40 mg/mL methoxyamine chloride in pyridine was added to the dried intracellular metabolites and incubated for 90 min at 30 °C and 200 rpm. The intracellular metabolites were then trimethylsilylated by adding 45 µL of *N*-methyl-*N*-trimethylsilyltrifluoroacetamide for 30 min at 37 °C and 200 rpm. The derivatized metabolite samples were analyzed by GC/MS by an Agilent 7890A GC/5975C MSD system equipped with an RTX-5Sil MS capillary column (30 m × 0.25 mm, 0.25 µm film thickness; Restek) and an additional 10 m long integrated guard column. One microliter of the derivatized sample was injected into the GC in a splitless mode. The oven temperature of a GC was initially set at 150 °C for 1 min, after which the temperature was increased to 330 °C at 20 °C/min, where it was held for 5 min. The mass spectra were recorded in a scan range 85–500 *m/z* at 70 eV of electron impact, and the temperature of the ion source and transfer line was 230 °C and 280 °C, respectively.

The raw data obtained from GC/MS analysis were processed using an automated mass spectral deconvolution and identification system (AMDIS) software for peak detection and deconvolution of mass spectra [57]. The processed data were uploaded to SpectConnect (http://spectconnect.mit.edu) for peak alignment and generating the data matrix with the Golm Metabolome Database (GMD) mass spectral reference library [58, 59]. The normalized abundance values for each metabolite were obtained by dividing the peak intensity with the dry cell weight. For statistical analysis, such as PCA and hierarchical cluster analysis representing as a heat map, the ClustVis Web tool was used [60].

## Supplementary information

**Additional file 1: Figure S1.** HPLC analysis of galactitol production with 20 g/L of galactose as the carbon source in nitrogen-rich medium after 48 h of growth: (a) 10 mM of galactose standard, (b) 10 mM of galactitol standard, and (c) 10× diluted test sample (20 g/L of galactose in rich medium) after 48 h of growth by *R. toruloides* IFO0880 showing galactitol production. Retention time is plotted on the *x*-axis, and galactose and galactitol intensities are plotted on the *y*-axis.

**Additional file 2: Figure S2.** Gas chromatography–mass spectrometry analysis of sample peaks. (a) Gas chromatogram showing peaks for galactose and galactitol, and (b) extracted mass spectra for galactose, and (c) extracted mass spectra for galactitol.

**Additional file 3: Figure S3.** Proton nuclear magnetic resonance (^1^H-NMR) spectroscopy analysis of galactitol. A total of 4 spectra are shown in Figure (all samples were dissolved in 90% H_2_O and 10% D_2_O). Panel (a) contains galactitol as the reference spectrum. Peaks at 3.84 ppm and 3.55 ppm were observed, noted with * symbol. Panel (b) shows the ^1^H spectrum of the culture media only. No galactitol signals were detected. Panel (c) contains the ^1^H spectrum of the product. The signals from galactitol show up clearly in this spectrum. Panel (d) is the spectrum collected after a few mg of galactitol powder were added directly to NMR tube (c). The signals from galactitol increased significantly, again indicating that the peaks in (c) are from galactitol.

**Additional file 4: Figure S4.** Growth of *R. toruloides* IFO0880 on different concentrations of galactitol in nitrogen-rich medium: (a) effect of galactitol on cell density, (b) utilization of galactitol. Circles, triangles, inverted triangles, diamonds are used to denote 0, 1, 5, and 10 g/L galactitol concentrations, respectively.

**Additional file 5: Figure S5.** Determination of molecular mass of AldR by SDS-PAGE. Lane M: molecular standard marker, Lane UI: uninduced crude extract, soluble fraction, Lane I: induced crude extract soluble fraction, and Lane P: purified AldR.

**Additional file 6: Figure S6.** Characterization of AldR from *R. toruloides* IFO0880 (a) Effect of pH on the activity of AldR. Enzyme assays were carried out under standard conditions in the presence of 10 mM galactose. Assays were carried out in 20 mM citrate buffer (pH 5–6) and 20 mM Tris–HCl buffer (pH 7–8). Activities at the optimal pH were defined as 100%. (b) Effect of temperature on the activity of AldR. Enzyme assays were carried out under standard conditions in the presence of 10 mM galactose in 20 mM Tris–HCl buffer (pH 7). Activities at the optimal temperature were defined as 100%.

**Additional file 7: Figure S7.** HPLC analysis of the reaction product obtained from in vitro reactions with galactose using AldR. The enzyme mixture containing 1 mg/mL AldR, 10 mM galactose, 10 mM NADPH, and 20 mM Tris–HCl buffer (pH 7.0) was incubated at 25 °C and 200 rpm for 16 h.

**Additional file 8: Figure S8.** A heat map of 29 intracellular metabolites in *R. toruloides* IFO0880 during growth on galactose versus glucose. The *x*-axis labels represent galactose and glucose as the carbon source, and *y*-axis labels represent the metabolites. All experiments were performed in triplicates.

**Additional file 9: Figure S9.** Intracellular metabolites present at higher concentrations during growth on galactose. The normalized abundance levels of the intracellular metabolites in *R. toruloides* IFO0880 grown on galactose (GAL) and glucose (GLC) are shown in box plots. The x-axis labels in the box plots represent the two different carbon sources and y-axis labels in the box plots represent the levels of metabolites.

**Additional file 10: Figure S10.** Intracellular metabolites present at higher concentrations during growth on glucose. The normalized abundance levels of the intracellular metabolites in *R. toruloides* IFO0880 grown on galactose (GAL) and glucose (GLC) are shown in box plots. The *x*-axis labels in the box plots represent the two different carbon sources and *y*-axis labels in the box plots represent the levels of metabolites.

**Additional file 11.** Tables S1–S5.

## Data Availability

The datasets used and/or analyzed during the current study are available from the corresponding author on reasonable request.

## Abbreviations

HPLC: high-performance liquid chromatography
GC–MS: gas chromatography–mass spectrometry
^1^H-NMR: proton nuclear magnetic resonance spectroscopy
AldR: aldose reductase
UT: galactose-1-phosphate uridyl transferase
TCA: citric acid cycle
XR: xylose reductase
XDH: xylitol dehydrogenase
YPD: yeast extract, peptone, and glucose
HPLC: high-performance liquid chromatography
ACT: actin gene
LB: Luria–Bertani medium

## References

[1] Grembecka M (2015). Sugar alcohols-their role in the modern world of sweeteners: a review. Eur Food Res Technol.

[2] Werpy T, Petersen G. Top value added chemicals from biomass: volume I—results of screening for potential candidates from sugars and synthesis gas. Golden: National Renewable Energy Lab; 2004.

[3] Livesey G (2007). Health potential of polyols as sugar replacers, with emphasis on low glycaemic properties. Nutr Res Rev.

[4] Jagtap S, Rao C (2018). Microbial conversion of xylose into useful bioproducts. Appl Microbiol Biotechnol.

[5] Jagtap S, Rao C (2018). Production of d-arabitol from d-xylose by the oleaginous yeast *Rhodosporidium toruloides* IFO0880. Appl Microbiol Biotechnol.

[6] Mirończuk A, Biegalska A, Dobrowolski A (2017). Functional overexpression of genes involved in erythritol synthesis in the yeast *Yarrowia lipolytica*. Biotechnol Biofuels.

[7] Mirończuk A, Rzechonek D, Biegalska A, Rakicka M, Dobrowolski A (2016). A novel strain of *Yarrowia lipolytica* as a platform for value-added product synthesis from glycerol. Biotechnol Biofuels.

[8] Moon H, Jeya M, Kim I, Lee J (2010). Biotechnological production of erythritol and its applications. Appl Microbiol Biotechnol.

[9] Pereira I, Madeira A, Prista C, Loureiro-Dias M, Leandro M (2014). Characterization of new polyol/H^+^ symporters in *Debaryomyces hansenii*. PLoS ONE.

[10] Saha B, Sakakibara Y, Cotta M (2007). Production of d-arabitol by a newly isolated *Zygosaccharomyces rouxii*. J Ind Microbiol Biotechnol.

[11] Corma A, Iborra S, Velty A (2007). Chemical routes for the transformation of biomass into chemicals. Chem Rev.

[12] Kordowska M (2015). Production of arabitol by yeasts: current status and future prospects. J Appl Microbiol.

[13] Rafiqul I, Sakinah A (2013). Processes for the production of xylitol—a review. Food Rev Int..

[14] Regnat K, Mach R, Mach-Aigner A (2018). Erythritol as sweetener—wherefrom and whereto?. Appl Microbiol Biotechnol.

[15] Rzechonek D, Dobrowolski A, Rymowicz W, Mirończuk A (2018). Recent advances in biological production of erythritol. Crit Rev Biotechnol.

[16] Coradetti S, Pinel D, Geiselman G, Ito M, Mondo S, Reilly M, Cheng Y, Bauer S, Grigoriev I, Gladden J (2018). Functional genomics of lipid metabolism in the oleaginous yeast *Rhodosporidium toruloides*. Elife..

[17] Fei Q, O’Brien M, Nelson R, Chen X, Lowell A, Dowe N (2016). Enhanced lipid production by *Rhodosporidium toruloides* using different fed-batch feeding strategies with lignocellulosic hydrolysate as the sole carbon source. Biotechnol Biofuels.

[18] Huang X, Liu J, Lu L, Peng K, Yang G, Liu J (2016). Culture strategies for lipid production using acetic acid as sole carbon source by *Rhodosporidium toruloides*. Bioresour Technol.

[19] Wiebe M, Koivuranta K, Penttilä M, Ruohonen L (2012). Lipid production in batch and fed-batch cultures of *Rhodosporidium toruloides* from 5 and 6 carbon carbohydrates. BMC Biotechnol.

[20] Zhang S, Ito M, Skerker J, Arkin A, Rao C (2016). Metabolic engineering of the oleaginous yeast *Rhodosporidium toruloides* IFO0880 for lipid overproduction during high-density fermentation. Appl Microbiol Biotechnol.

[21] Zhang S, Skerker J, Rutter C, Maurer M, Arkin A, Rao C (2015). Engineering *Rhodosporidium toruloides* for increased lipid production. Biotechnol Bioeng.

[22] Yaegashi J, Kirby J, Ito M, Sun J, Dutta T, Mirsiaghi M, Sundstrom E, Rodriguez A, Baidoo E, Tanjore D (2017). *Rhodosporidium toruloides*: a new platform organism for conversion of lignocellulose into terpene biofuels and bioproducts. Biotechnol Biofuels.

[23] Lee J, Chen L, Cao B, Chen W (2016). Engineering *Rhodosporidium toruloides* with a membrane transporter facilitates production and separation of carotenoids and lipids in a bi-phasic culture. Appl Microbiol Biotechnol.

[24] Lee J, Chen L, Shi J, Trzcinski A, Chen W (2014). Metabolomic profiling of *Rhodosporidium toruloides* grown on glycerol for carotenoid production during different growth phases. J Agric Food Chem..

[25] Zhuang X, Kilian O, Monroe E, Ito M, Tran-Gymfi MB, Liu F, Davis R, Mirsiaghi M, Sundstrom E, Pray T (2019). Monoterpene production by the carotenogenic yeast *Rhodosporidium toruloides*. Microb Cell Fact..

[26] Wehrs M, Gladden J, Liu Y, Platz L, Prahl JP, Moon J, Papa G, Sundstrom E, Geiselman G, Tanjore D (2019). Sustainable bioproduction of the blue pigment indigoidine: expanding the range of heterologous products in *R. toruloides* to include non-ribosomal peptides. Green Chem..

[27] Ageitos J, Vallejo J, Veiga-Crespo P, Villa T (2011). Oily yeasts as oleaginous cell factories. Appl Microbiol Biotechnol.

[28] Park Y, Nicaud J, Ledesma-Amaro R (2018). The engineering potential of *Rhodosporidium toruloides* as a workhorse for biotechnological applications. Trends Biotechnol.

[29] Zhu Z, Zhang S, Liu H, Shen H, Lin X, Yang F, Zhou Y, Jin G, Ye M, Zou H (2012). A multi-omic map of the lipid-producing yeast *Rhodosporidium toruloides*. Nat Commun..

[30] Dien B, Slininger P, Kurtzman C, Moser B, O’Bryan P (2016). Identification of superior lipid producing *lipomyces* and *myxozyma* yeasts. AIMS Environ Sci.

[31] Natarajan J, Madras G, Chatterjee K (2017). Development of graphene oxide-/galactitol polyester-based biodegradable composites for biomedical applications. ACS Omega..

[32] Natarajan J, Movva S, Madras G, Chatterjee K (2017). Biodegradable galactitol based crosslinked polyesters for controlled release and bone tissue engineering. Mater Sci Eng C Mater Biol Appl..

[33] Clark J, Livesey J, Steele J (1996). Delay or inhibition of rat lens opacification using pantethine and WR-77913. Exp Eye Res.

[34] Jiang X, Huang Y, Wang X, Liang Q, Li Y, Li F, Fu X, Huang C, Liu H (2017). Dianhydrogalactitol, a potential multitarget agent, inhibits glioblastoma migration, invasion, and angiogenesis. Biomed Pharmacother.

[35] Kamada M, Satoh T, Yokota K, Kakuchi T (1999). Regio- and stereoselective cyclopolymerization of 1,2:5,6-dianhydroallitol and 1,2:5,6-dianhydrogalactitol leading to a novel carbohydrate polymer of (2 → 6)-1,5-anhydro-dl-galactitol. Macromolecules.

[36] Zhang X, Lian Y, Guo W, Xu B, Li M, Zhou Y, Rong C (2009). Anticancer activity and mechanisms of diacetyldianhydrogalactitol on hepatoma QGY-7703 cells. Anticancer Drug..

[37] Caputto R, Leloir L, Trucco R, Cardini C, Paladini A (1949). The enzymatic transformation of galactose into glucose derivatives. J Biol Chem.

[38] Lazar Z, Gamboa-Meléndez H, Coq A, Neuvéglise C, Nicaud J (2015). Awakening the endogenous Leloir pathway for efficient galactose utilization by *Yarrowia lipolytica*. Biotechnol Biofuels.

[39] Meyer J, Walker J, Hollenberg C (1991). Galactokinase encoded by GAL1 is a bifunctional protein required for induction of the GAL genes in *Kluyveromyces lactis* and is able to suppress the gal3 phenotype in *Saccharomyces cerevisiae*. Mol Cell Biol.

[40] Sellick C, Campbell R, Reece R (2008). Galactose metabolism in yeast-structure and regulation of the Leloir pathway enzymes and the genes encoding them. Int Rev Cell Mol Biol.

[41] Zaman S, Lippman S, Zhao X, Broach J (2008). How *Saccharomyces* responds to nutrients. Annu Rev Genet.

[42] Gancedo J (1998). Yeast carbon catabolite repression. Microbiol Mol Biol Rev.

[43] Kuhn A, Zyl C, Tonder A, Prior B (1995). Purification and partial characterization of an aldo-keto reductase from *Saccharomyces cerevisiae*. Appl Environ Microbiol.

[44] Liu J, Zhang G, Kong I, Yun E, Zheng J, Kweon D, Jin Y (2018). A Mutation in PGM2 causing inefficient galactose metabolism in the probiotic yeast *Saccharomyces boulardii*. Appl Environ Microbiol.

[45] Petrash J (2004). All in the family: aldose reductase and closely related aldo-keto reductases. Cell Mol Life Sci.

[46] de Jongh W, Bro C, Ostergaard S, Regenberg B, Olsson L, Nielsen J (2008). The roles of galactitol, galactose-1-phosphate, and phosphoglucomutase in galactose-induced toxicity in *Saccharomyces cerevisiae*. Biotechnol Bioeng.

[47] Los E, Ford G. Galactose-1-phosphate uridyltransferase deficiency (galactosemia). In: StatPearls. StatPearls Publishing. 2017. 28722986

[48] Muniruzzaman S, Itoh H, Yoshino A, Katayama T, Izumori K (1994). Biotransformation of lactose to galactitol. J Ferment Bioeng.

[49] Onishi H, Suzuki T (1968). Formation of dulcitol in aerobic dissimilation of d-galactose by yeasts. J Bacteriol.

[50] Masuda C, Previato J, Miranda M, Assis L, Penha L, Mendonça-Previato L, Montero-Lomelí M (2008). Overexpression of the aldose reductase GRE3 suppresses lithium-induced galactose toxicity in *Saccharomyces cerevisiae*. FEMS Yeast Res.

[51] Yun E, Oh E, Liu J, Yu S, Kim D, Kwak S, Kim K, Jin Y (2018). Promiscuous activities of heterologous enzymes lead to unintended metabolic rerouting in *Saccharomyces cerevisiae* engineered to assimilate various sugars from renewable biomass. Biotechnol Biofuels.

[52] Liu J, Zhang G, Kwak S, Oh E, Yun E, Chomvong K, Cate J, Jin Y (2019). Overcoming the thermodynamic equilibrium of an isomerization reaction through oxidoreductive reactions for biotransformation. Nat Commun..

[53] Izard J, Limberger R (2003). Rapid screening method for quantitation of bacterial cell lipids from whole cells. J Microbiol Methods.

[54] Quarterman J, Slininger P, Kurtzman C, Thompson S, Dien B (2017). A survey of yeast from the *Yarrowia* clade for lipid production in dilute acid pretreated lignocellulosic biomass hydrolysate. Appl Microbiol Biotechnol.

[55] Lepage G, Roy C (1986). Direct transesterification of all classes of lipids in a one-step reaction. J Lipid Res.

[56] Kim S, Lee D, Wohlgemuth G, Park H, Fiehn O, Kim K (2013). Evaluation and optimization of metabolome sample preparation methods for *Saccharomyces cerevisiae*. Anal Chem.

[57] Stein S (1999). An integrated method for spectrum extraction and compound identification from gas chromatography/mass spectrometry data. J Am Soc Mass Spectrom.

[58] Kopka J, Schauer N, Krueger S, Birkemeyer C, Usadel B, Bergmüller E, Dörmann P, Weckwerth W, Gibon Y, Stitt M (2005). GMD@CSB.DB: the Golm Metabolome Database. Bioinformatics..

[59] Styczynski M, Moxley J, Tong L, Walther J, Jensen K, Stephanopoulos G (2007). Systematic identification of conserved metabolites in GC/MS data for metabolomics and biomarker discovery. Anal Chem.

[60] Metsalu T, Vilo J (2015). ClustVis: a web tool for visualizing clustering of multivariate data using principal component analysis and heatmap. Nucleic Acids Res.

