# Supplementary figures and images for: Production of galactitol from galactose by the oleaginous yeast Rhodosporidium toruloides IFO0880
Source: Biotechnol Biofuels. 2019 Oct 18;12:250. doi: 10.1186/s13068-019-1586-5 (PMC6798376; doi:10.1186/s13068-019-1586-5)

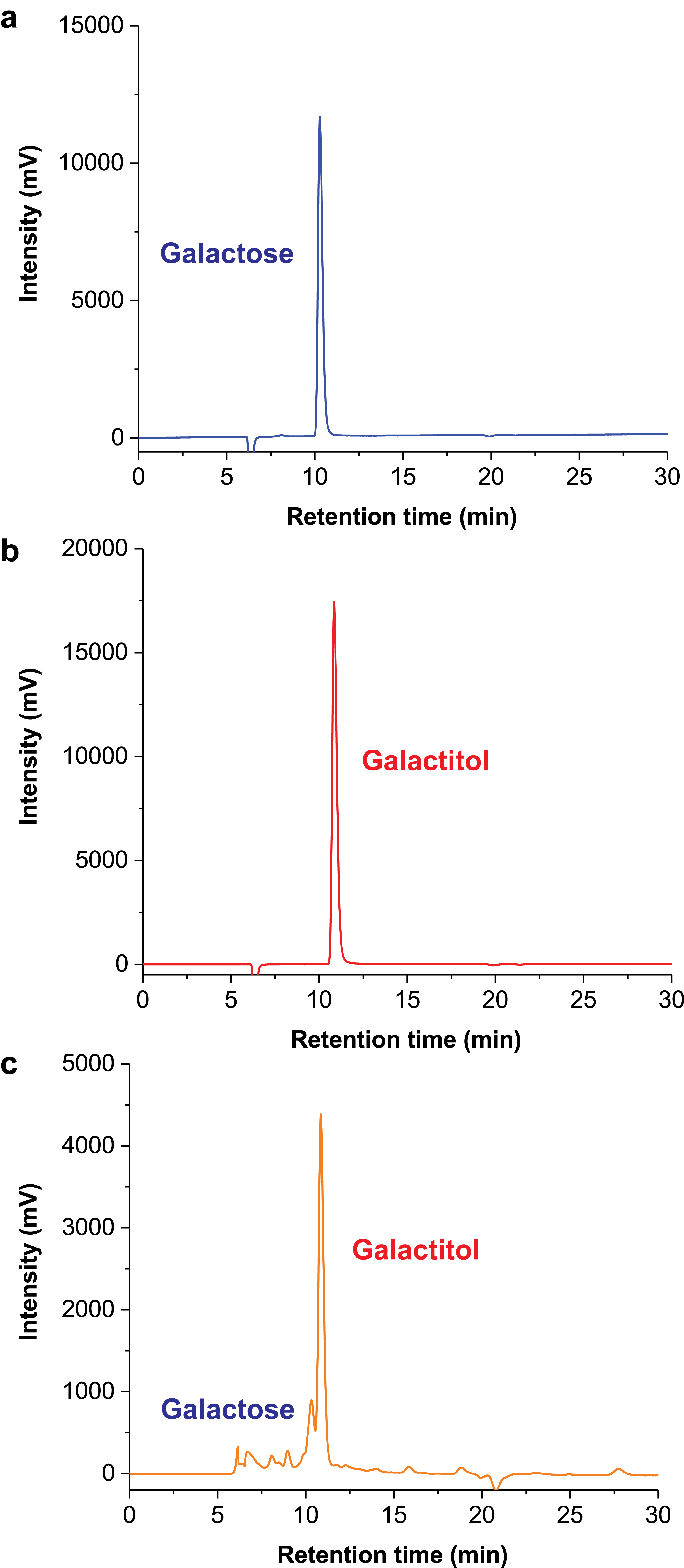

Supplement: Supplementary file 1 — Additional file 1: Figure S1. HPLC analysis of galactitol production with 20 g/L of galactose as the carbon source in nitrogen-rich medium after 48 h of growth: (a) 10 mM of galactose standard, (b) 10 mM of galactitol standard, and (c) 10× diluted test sample (20 g/L of galactose in rich medium) after 48 h of growth by R. toruloides IFO0880 showing galactitol production. Retention time is plotted on the x-axis, and galactose and galactitol intensities are plotted on the y-axis. [file 13068_2019_1586_MOESM1_ESM.tif]

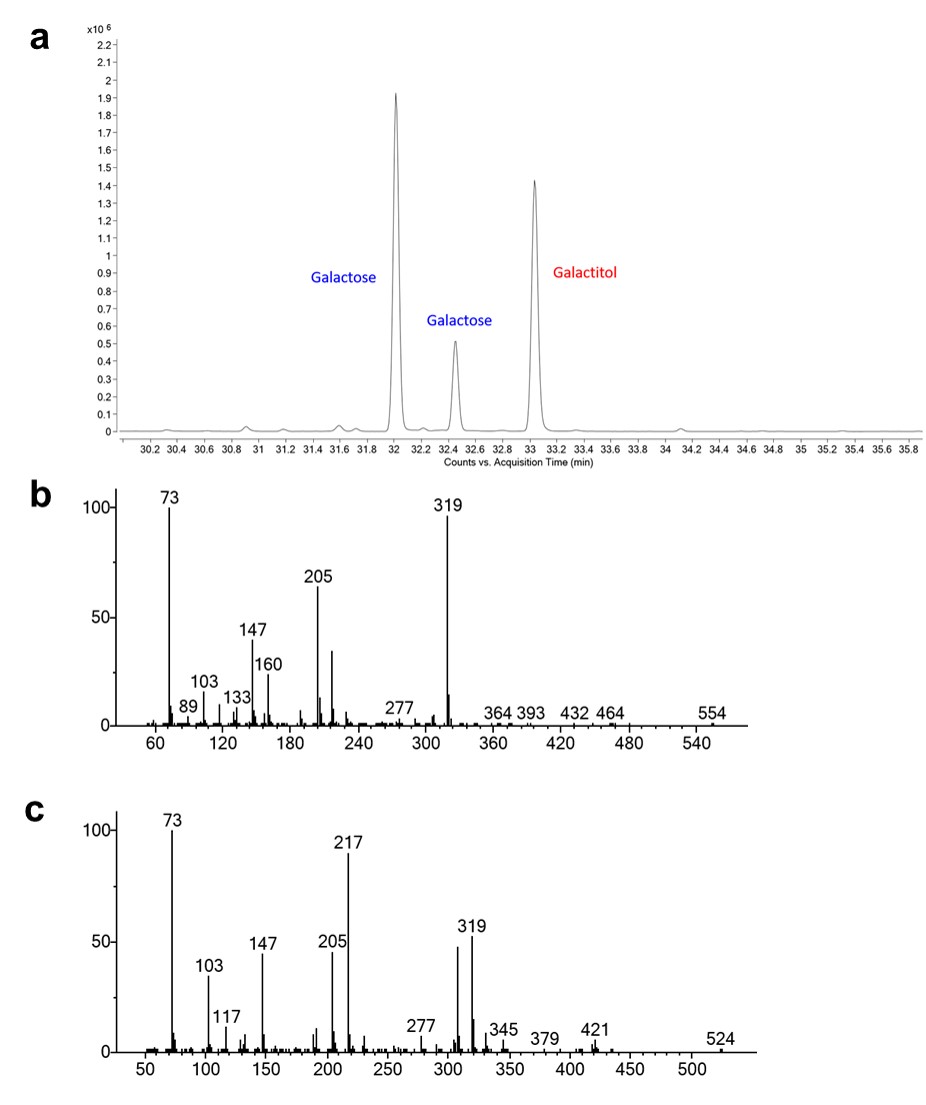

Supplement: Supplementary file 2 — Additional file 2: Figure S2. Gas chromatography–mass spectrometry analysis of sample peaks. (a) Gas chromatogram showing peaks for galactose and galactitol, and (b) extracted mass spectra for galactose, and (c) extracted mass spectra for galactitol. [file 13068_2019_1586_MOESM2_ESM.jpg]

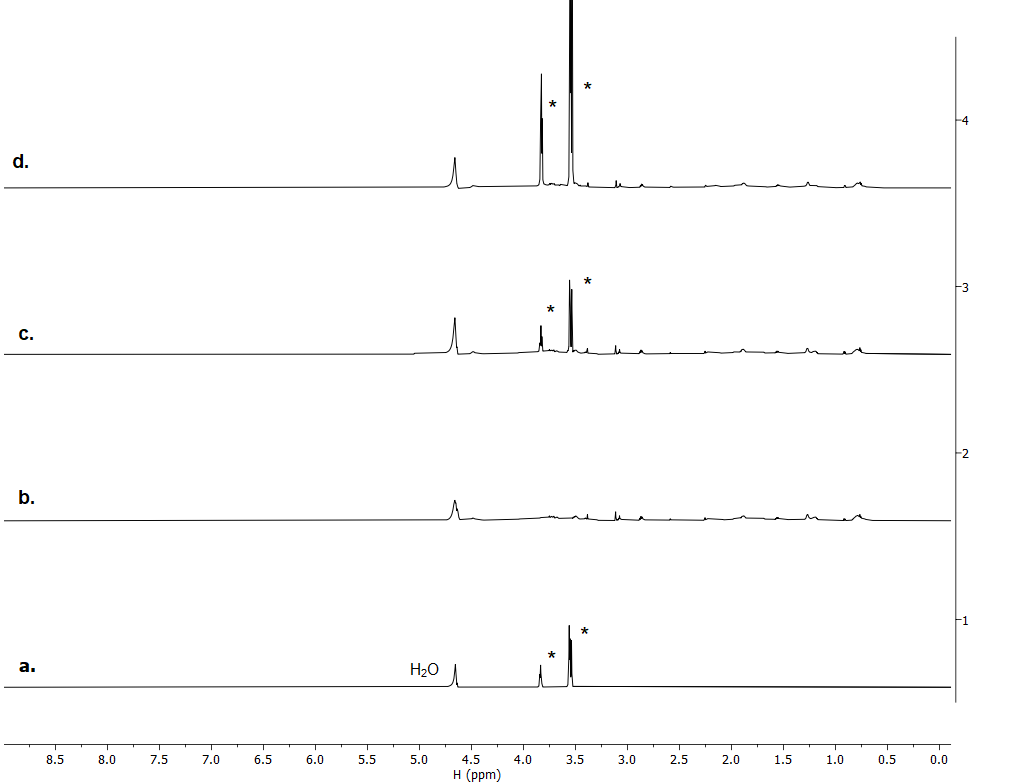

Supplement: Supplementary file 3 — Additional file 3: Figure S3. Proton nuclear magnetic resonance (1H-NMR) spectroscopy analysis of galactitol. A total of 4 spectra are shown in Figure (all samples were dissolved in 90% H2O and 10% D2O). Panel (a) contains galactitol as the reference spectrum. Peaks at 3.84 ppm and 3.55 ppm were observed, noted with * symbol. Panel (b) shows the 1H spectrum of the culture media only. No galactitol signals were detected. Panel (c) contains the 1H spectrum of the product. The signals from galactitol show up clearly in this spectrum. Panel (d) is the spectrum collected after a few mg of galactitol powder were added directly to NMR tube (c). The signals from galactitol increased significantly, again indicating that the peaks in (c) are from galactitol. [file 13068_2019_1586_MOESM3_ESM.tiff]

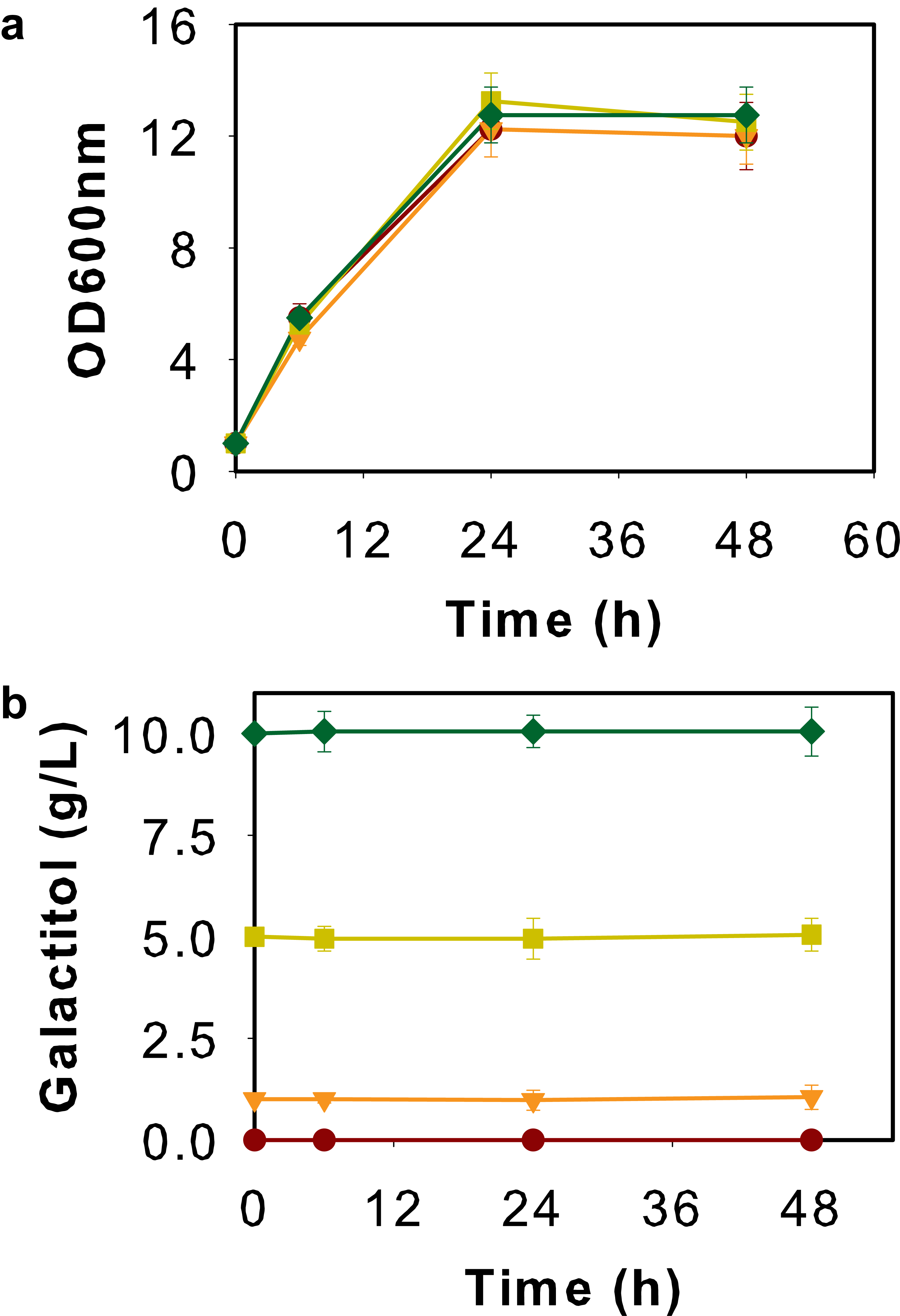

Supplement: Supplementary file 4 — Additional file 4: Figure S4. Growth of R. toruloides IFO0880 on different concentrations of galactitol in nitrogen-rich medium: (a) effect of galactitol on cell density, (b) utilization of galactitol. Circles, triangles, inverted triangles, diamonds are used to denote 0, 1, 5, and 10 g/L galactitol concentrations, respectively. [file 13068_2019_1586_MOESM4_ESM.tif]

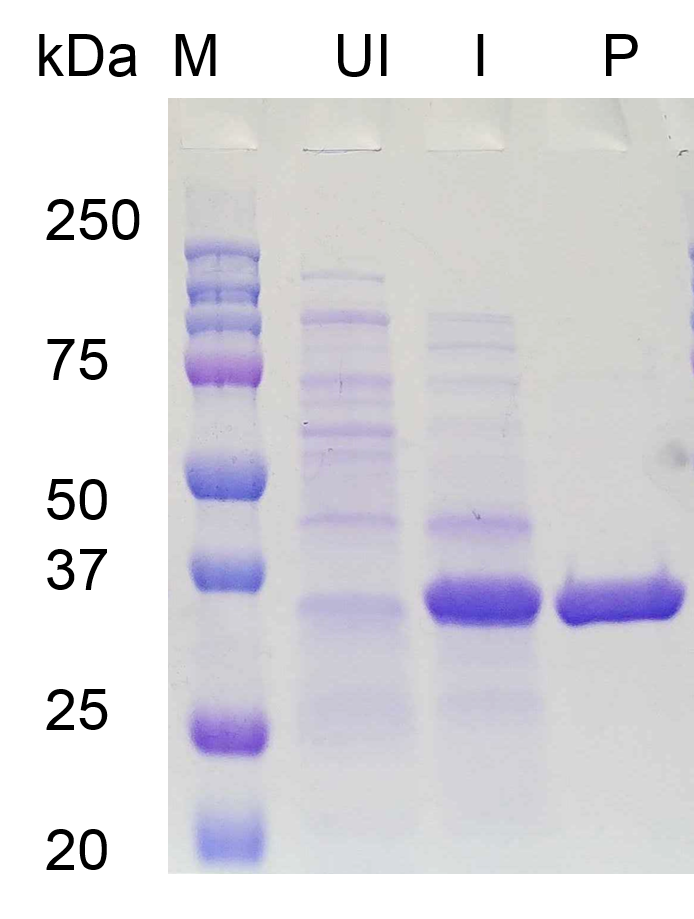

Supplement: Supplementary file 5 — Additional file 5: Figure S5. Determination of molecular mass of AldR by SDS-PAGE. Lane M: molecular standard marker, Lane UI: uninduced crude extract, soluble fraction, Lane I: induced crude extract soluble fraction, and Lane P: purified AldR. [file 13068_2019_1586_MOESM5_ESM.tif]

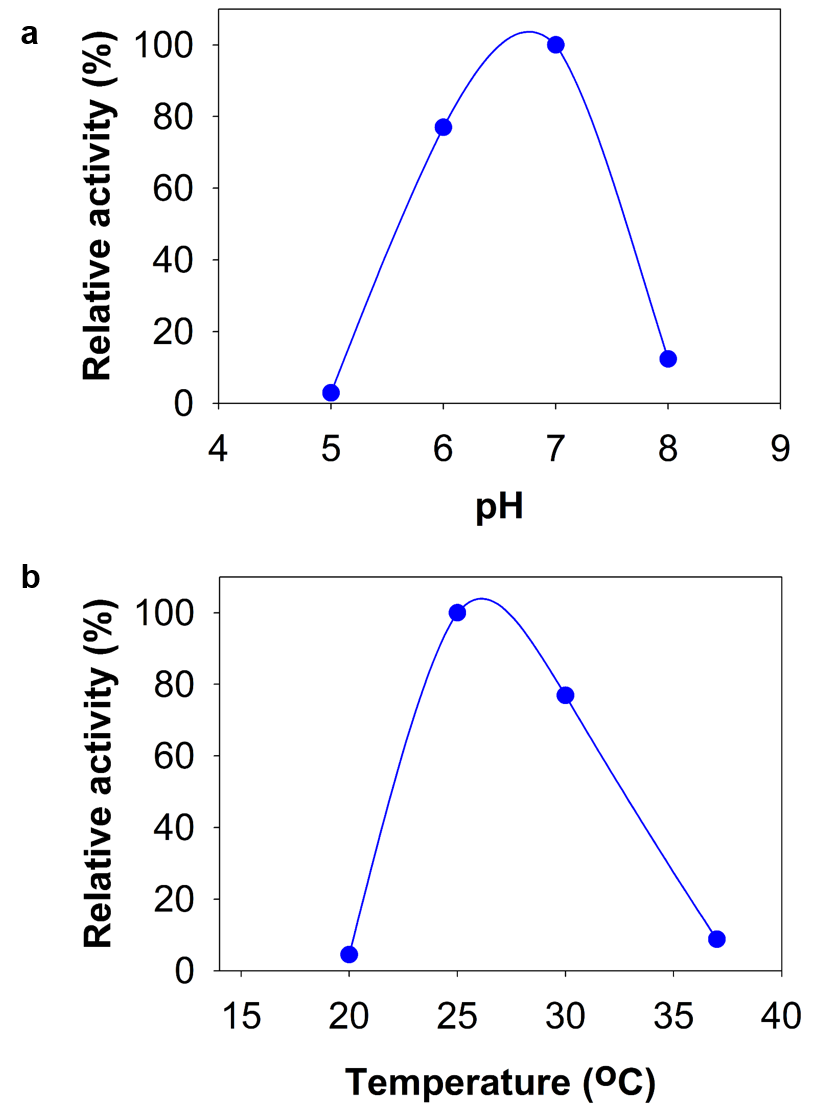

Supplement: Supplementary file 6 — Additional file 6: Figure S6. Characterization of AldR from R. toruloides IFO0880 (a) Effect of pH on the activity of AldR. Enzyme assays were carried out under standard conditions in the presence of 10 mM galactose. Assays were carried out in 20 mM citrate buffer (pH 5–6) and 20 mM Tris–HCl buffer (pH 7–8). Activities at the optimal pH were defined as 100%. (b) Effect of temperature on the activity of AldR. Enzyme assays were carried out under standard conditions in the presence of 10 mM galactose in 20 mM Tris–HCl buffer (pH 7). Activities at the optimal temperature were defined as 100%. [file 13068_2019_1586_MOESM6_ESM.tif]

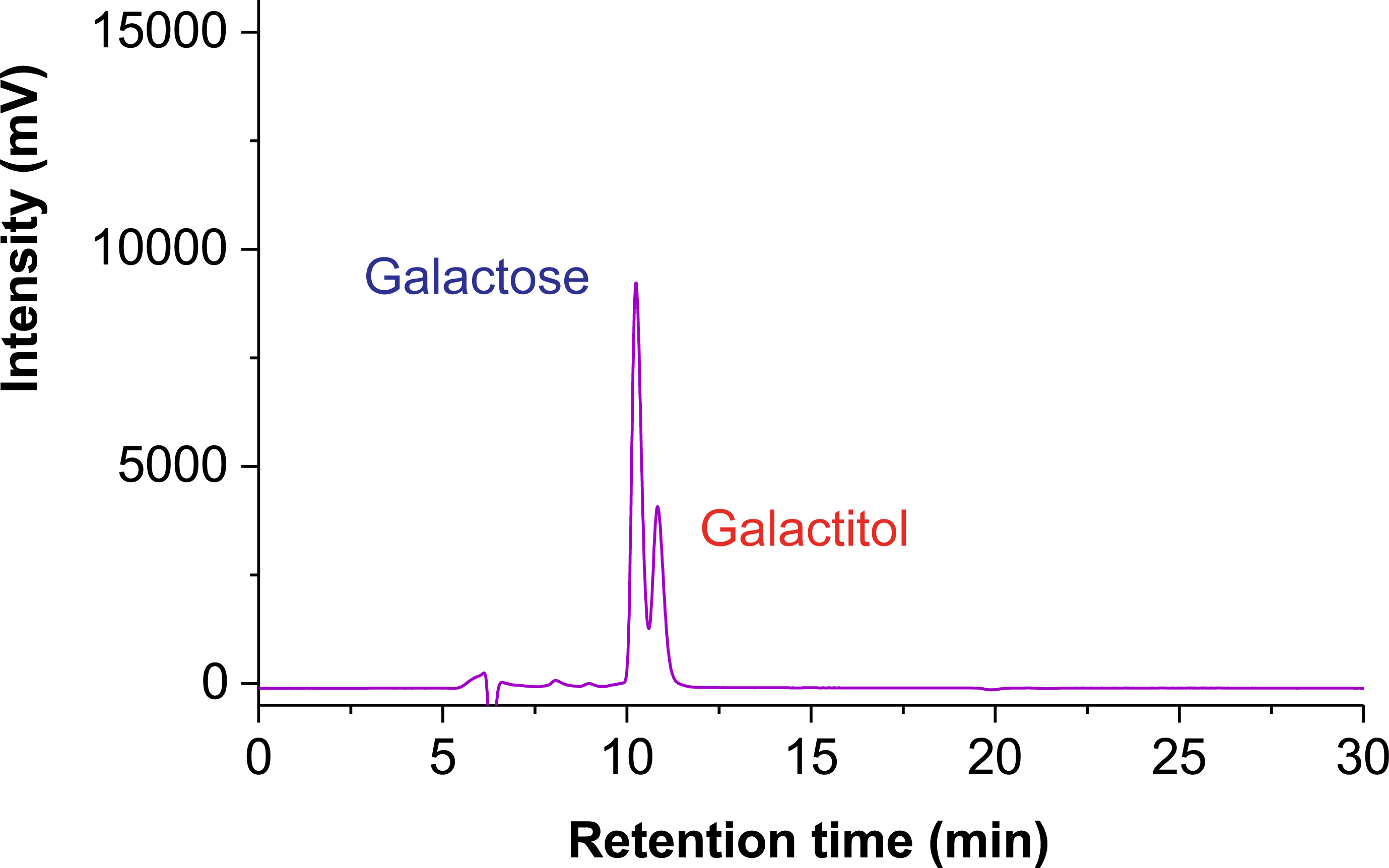

Supplement: Supplementary file 7 — Additional file 7: Figure S7. HPLC analysis of the reaction product obtained from in vitro reactions with galactose using AldR. The enzyme mixture containing 1 mg/mL AldR, 10 mM galactose, 10 mM NADPH, and 20 mM Tris–HCl buffer (pH 7.0) was incubated at 25 °C and 200 rpm for 16 h. [file 13068_2019_1586_MOESM7_ESM.tif]

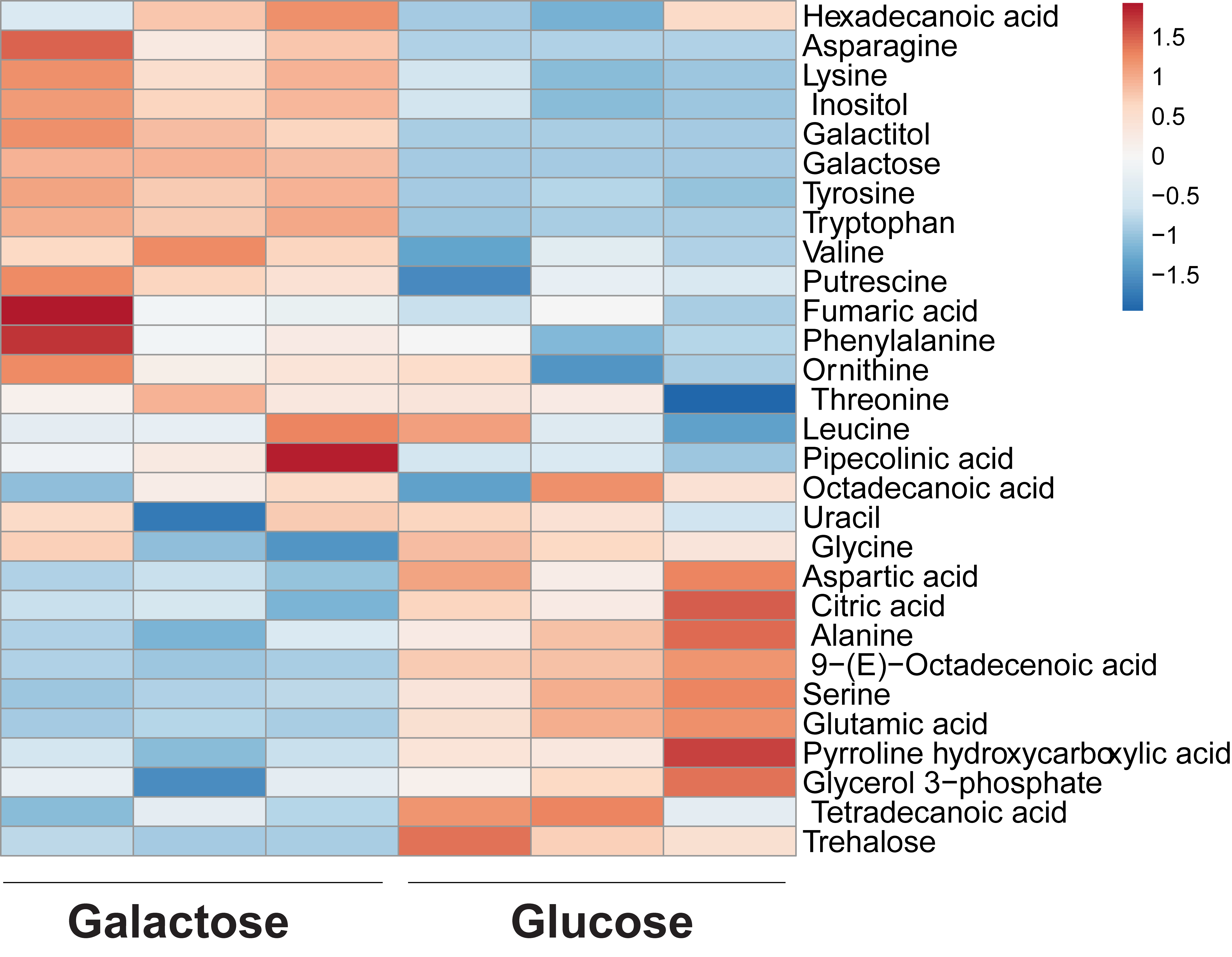

Supplement: Supplementary file 8 — Additional file 8: Figure S8. A heat map of 29 intracellular metabolites in R. toruloides IFO0880 during growth on galactose versus glucose. The x-axis labels represent galactose and glucose as the carbon source, and y-axis labels represent the metabolites. All experiments were performed in triplicates. [file 13068_2019_1586_MOESM8_ESM.tif]

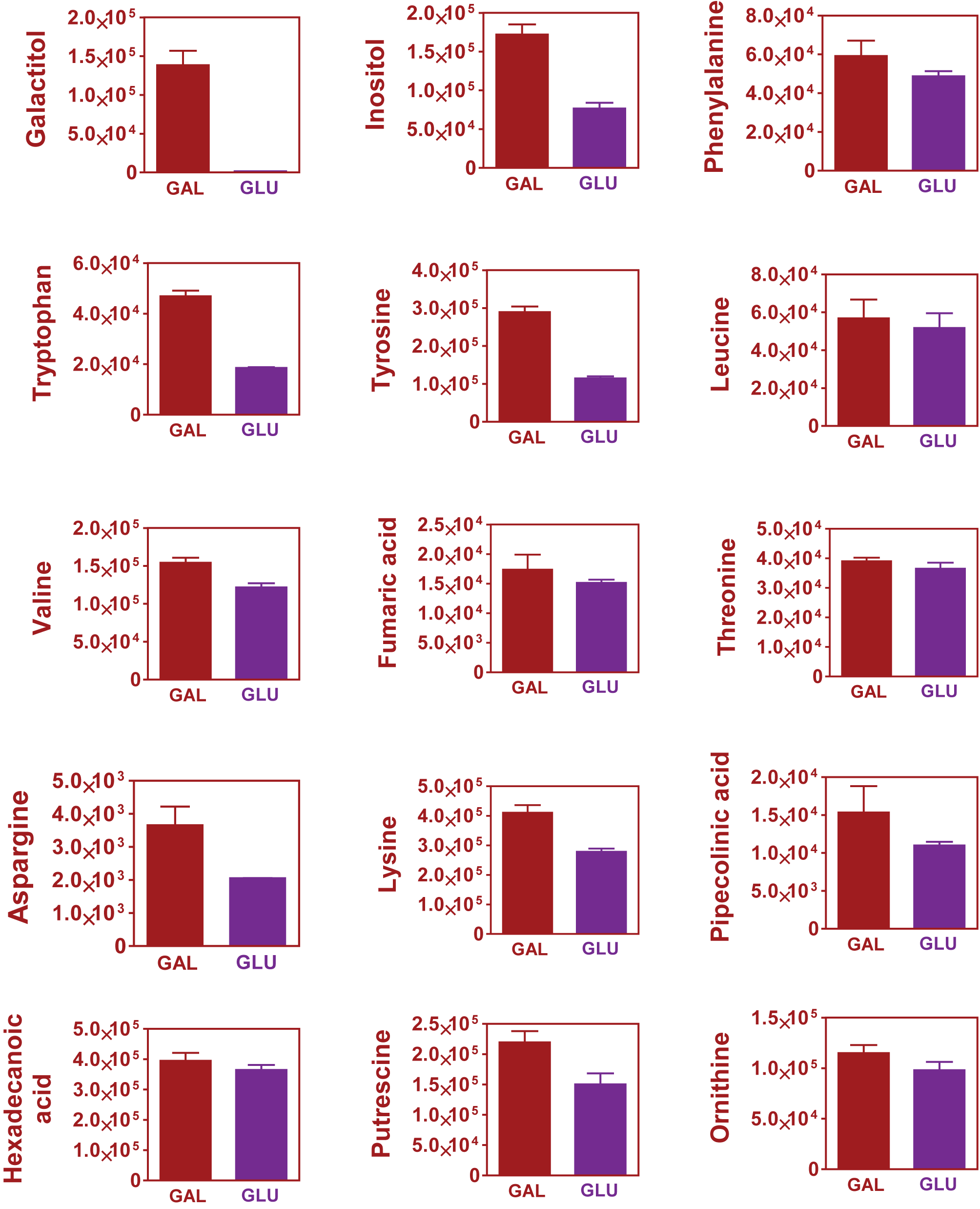

Supplement: Supplementary file 9 — Additional file 9: Figure S9. Intracellular metabolites present at higher concentrations during growth on galactose. The normalized abundance levels of the intracellular metabolites in R. toruloides IFO0880 grown on galactose (GAL) and glucose (GLC) are shown in box plots. The x-axis labels in the box plots represent the two different carbon sources and y-axis labels in the box plots represent the levels of metabolites. [file 13068_2019_1586_MOESM9_ESM.tif]

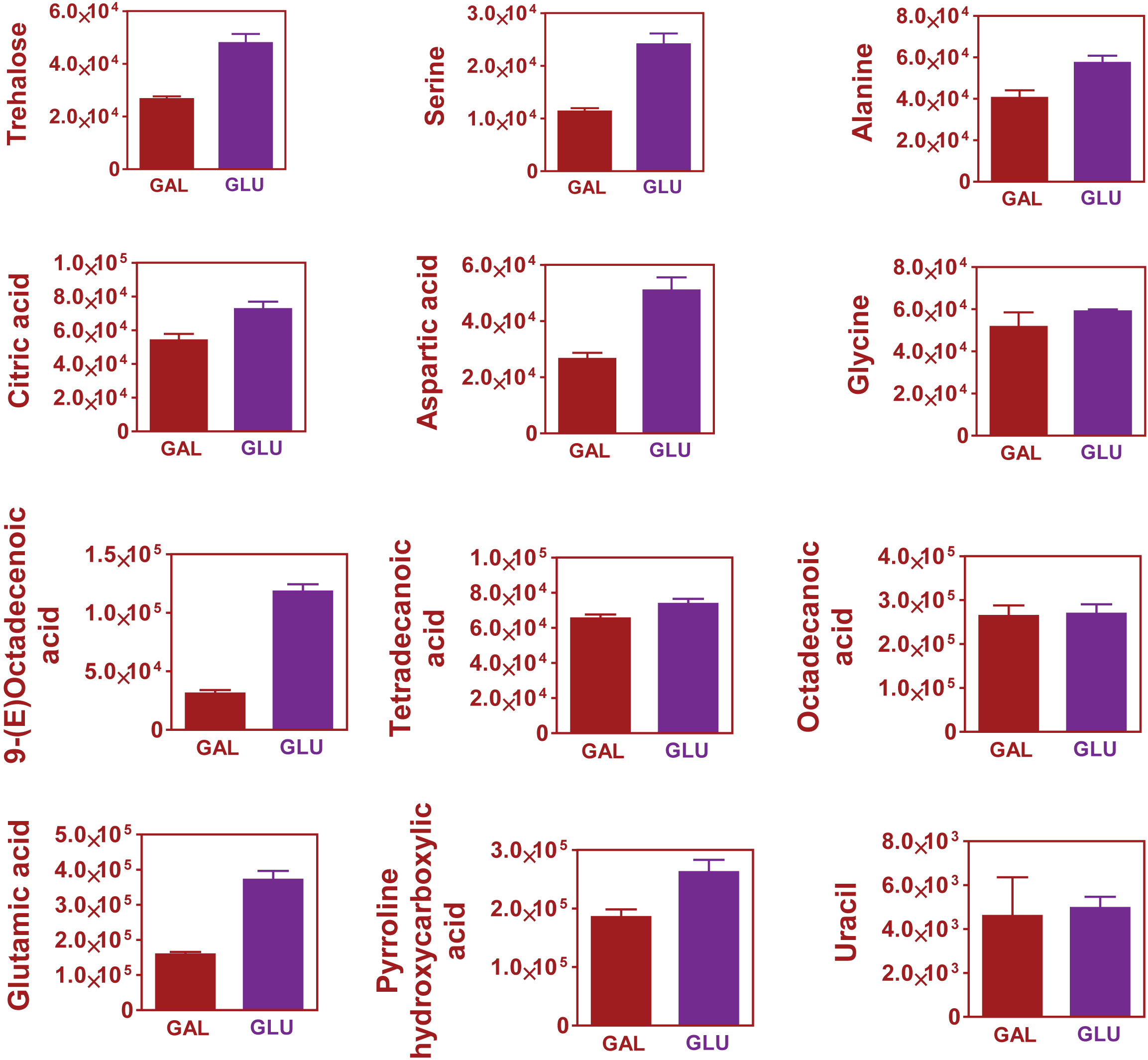

Supplement: Supplementary file 10 — Additional file 10: Figure S10. Intracellular metabolites present at higher concentrations during growth on glucose. The normalized abundance levels of the intracellular metabolites in R. toruloides IFO0880 grown on galactose (GAL) and glucose (GLC) are shown in box plots. The x-axis labels in the box plots represent the two different carbon sources and y-axis labels in the box plots represent the levels of metabolites. [file 13068_2019_1586_MOESM10_ESM.tif]
