# Supplementary material for: Production of galactitol from galactose by the oleaginous yeast Rhodosporidium toruloides IFO0880
Source: Biotechnol Biofuels. 2019 Oct 18;12:250. doi: 10.1186/s13068-019-1586-5 (PMC6798376; doi:10.1186/s13068-019-1586-5)
Supplement: Supplementary file 11 — Additional file 11. Tables S1–S5. [file 13068_2019_1586_MOESM11_ESM.docx]

**ADDITIONAL INFORMATION FOR**

**Production of galactitol from galactose by the oleaginous yeast *Rhodosporidium toruloides* IFO0880**

Sujit Sadashiv Jagtap^13^, Ashwini Ashok Bedekar^1^, Jing-Jing Liu^3^, Yong-Su Jin^23^, and Christopher V. Rao*^13^

^1^Department of Chemical and Biomolecular Engineering

^2^Department of Food Science and Nutrition

^3^DOE Center for Advanced Bioenergy and Bioproducts Innovation

University of Illinois at Urbana-Champaign

600 S. Mathews Ave., Urbana, IL 61801, USA.

*Corresponding author.

E-mail: cvrao@illinois.edu. Phone: (217) 244-2247. Fax: (217) 333-5052

**Table S1.** Comparison of galactitol production rate and yield using galactose in nitrogen rich medium (YP) and low nitrogen medium (LNM) by *R. toruloides*.

| **Medium** | **Galactose (g/L)** | **Galactitol production rate (g/L h)** | **Galactitol yield**  **(g/g sugar)** |
| --- | --- | --- | --- |
| YP | 20 | 0.046 ± 0.004 | 0.165 ± 0.015 |
| YP | 40 | 0.061 ± 0.005 | 0.220 ± 0.018 |
| YP | 60 | 0.061 ± 0.006 | 0.147 ± 0.015 |
| LNM | 20 | 0.021 ± 0.003 | 0.150 ± 0.020 |
| LNM | 40 | 0.028 ± 0.003 | 0.136 ± 0.017 |
| LNM | 60 | 0.036 ± 0.006 | 0.114 ± 0.018 |

**Table S2**. Oligonucleotides used in this study.

| **Primer** | **Sequence** | **Characteristic** |
| --- | --- | --- |
| SJaldRcoFP | C GTT AAT GTG GAA TAGAAGCTTGCGGCCGCACTC | PET28a-PCR |
| SJaldRcoRP | GTT CGG CCC CCA TGG ATC CGC GAC CCA TTT GC | PET28a-PCR |
| SJaldRco-pet28aFP | GACATATCTA GGA TCC ATG GGG GCC GAA C | aldRco-PCR |
| SJaldRco-pet28aRP | CAG ATG AGA TAA GCT TCT ATT CCA CAT TAA CGG GAT TCC AG | aldRco-PCR |
| SJgalmut qPCR FWD | GCT GAA CCA CCA TCT CTT CAT | GM, RT-PCR |
| SJgalmut qPCR REV | CCT TCT TGA TTG GGT CGA TCT T | GM, RT-PCR |
| AldR FWD | TCG TCG AAC TCT CCA AGA AAC | AldR, RT-PCR |
| AldR REV | CTC TTC GGT AGC GGA ACA AA | AldR, RT-PCR |
| GK FWD | CAT CGA ATT CAT CCC GAC TCT C | GK, RT-PCR |
| GK REV | ACG CAG GTT GTA GTG GTA TTT | GK, RT-PCR |
| UT FWD | TGC CGA GTT TGG CGT ATT | UT, RT-PCR |
| UT REV | AGG AGC ATT CGA AGA GGT TAT C | UT, RT-PCR |
| EP FWD | GAC AAC TAC CAC AAC TCC TTC C | EP, RT-PCR |
| EP REV | CAA TCT CGA GCG CCT TCT T | EP, RT-PCR |
| PGM FWD | CCA AGA CGA TCA AGG AGT ACA A | PGM, RT-PCR |
| PGM REV | CGC GTC GAT AAC CTC AAT CT | PGM, RT-PCR |
| ActinFP | CCTCTACGGCAACATTGTCA | Act, RT-PCR |
| ActinRP | TTCGAGATCCACATCTGCTG | Act, RT-PCR |

**Table S3**. Nucleotide sequence of codon optimized aldose reductase.

| Name | Sequence (5’-3’) |
| --- | --- |
| AldR | ATG GGG GCC GAA CAT GCC GTA GCG TCT CCA TTC ACT CTT GCT AGC TCT GTT AAG CTG CGC AAC GGC GCC CAA ATG CCA CGC CTTGGT TTT GGT GTT TTC CAG TCA ACC AAC GCT AAA GCT AGT ACG GCA CAT GCA TTG ACG ATG GGA TAC CGC CAT ATA GAT TCT GCTCGC TAC TAC CAC AAC GAA GAG GAA GTG TGC GCA GCA GTC CAA AAG TTT TCC GGT GGG AAT CTG CCC AAC GAA GGG ACC GGT AAAGTC TGG CTG ACC ACC AAG GTC ATG GGG CAG GAA CAT GGT ACG GAC CAA ACT AAC AAG GCG GTT GAT GAA TCC GTG GCA ATA GCGAAA AAG TAC GGA CTG ACA TGG GAT TTA TTC CTG CTG CAT GAC CCT ACT GCA GGA AAG CAG AAG AGA CTT GAA GCC TGG AAG GTGCTT ATC GAG AAG CGG GAT CAA GGT CTG ATC AAA TCC ATT GGA GTT TCC AAC TTC GGT GTC AAG CAC CTT GAG CAG ATC AAG GAAGCA GGG TTA GAG ACA CCG GAG GTT AAT CAG ATT GAA CTG CAT CCT TTC TTA CAG CAG CGC GAC ATA GTC GAG TAT TGT GAG AAGGAG GGG ATC GTT GTC GAA GCA TAT TGT CCA ATT TTG CGG GGT AAG CGG TTT GAC GAT CCT ACA CTG GTC GAA CTT AGC AAG AAACAT TCC GTG ACC GTG CCC CAG ATC CTT ATT AGA TGG TCA TTG CAA AAG GGG TTT GTC CCA CTG CCG AAA TCA GAC ACT CCT GGACGT ATT CAA GCC AAC GCG GAT TTA TGG GAT TTC GAG CTT GAT GAG GGC GAT ATG CAA CAA ATG GAG AAA TTA GAT GAG GGT TATGCG GTA AGC TGG AAT CCC GTT AAT GTG GAA TAG |

**Table S4** A total of 29 significant intracellular metabolites such as amino acids, sugars, phosphates, fatty acids, organic acids, nucleosides and others, identified under glucose and galactose conditions by GC-MS analysis in *R. toruloides* IFO0880.

| Sr. No. | Metabolite name |
| --- | --- |
| 1 | Leucine |
| 2 | Valine |
| 3 | Serine |
| 4 | Threonine |
| 5 | Glycine |
| 6 | Uracil |
| 7 | Fumaric acid |
| 8 | Asparagine |
| 9 | Pyrroline hydroxycarboxylic acid |
| 10 | Aspartic acid |
| 11 | Pipecolinic acid |
| 12 | Glutamic acid |
| 13 | Phenylalanine |
| 14 | Putrescine |
| 15 | Glycerol 3-phosphate |
| 16 | Ornithine |
| 17 | Citric acid |
| 18 | Tetradecanoic acid |
| 19 | Galactose |
| 20 | Lysine |
| 21 | Tyrosine |
| 22 | Galactitol |
| 23 | Hexadecanoic acid |
| 24 | Inositol |
| 25 | 9-(E)-Octadecenoic acid |
| 26 | Tryptophan |
| 27 | Octadecanoic acid |
| 28 | Trehalose |
| 29 | Alanine |

**Table S5** Twenty nine metabolites with high absolute loadings as determined by PCA.

| **Sr. No.** | **Metabolite** | **Loading** | | | | | |
| --- | --- | --- | --- | --- | --- | --- | --- |
|  |  | PC1 | PC2 | PC3 | PC4 | PC5 | PC6 |
| 1 | Leucine | -0.0767 | -0.2126 | -0.4529 | -0.3191 | 0.0266 | 0.3778 |
| 2 | Valine | -0.2028 | 0.1826 | 0.0200 | 0.2021 | 0.0438 | 0.2642 |
| 3 | Serine | 0.2203 | 0.0800 | 0.0577 | -0.0037 | 0.1031 | -0.4814 |
| 4 | Threonine | -0.1322 | -0.1369 | -0.3940 | 0.3206 | 0.0009 | 0.0608 |
| 5 | Glycine | 0.1394 | -0.3425 | 0.2212 | 0.1859 | 0.0784 | -0.0855 |
| 6 | Uracil | 0.0151 | -0.3327 | -0.0955 | -0.3287 | 0.5273 | -0.0072 |
| 7 | Fumaric acid | -0.1413 | -0.2295 | 0.2849 | 0.2391 | 0.2727 | 0.0090 |
| 8 | Asparagine | -0.2116 | -0.0761 | 0.1652 | -0.0663 | 0.1169 | -0.1025 |
| 9 | Pyrroline  hydroxycarboxylic acid | 0.2038 | 0.0352 | 0.2040 | -0.2015 | 0.0008 | 0.2845 |
| 10 | Aspartic acid | 0.2131 | -0.0502 | 0.0623 | -0.0940 | -0.2286 | -0.0368 |
| 11 | Pipecolinic acid | -0.1646 | 0.1342 | -0.2647 | -0.3042 | 0.2049 | -0.2341 |
| 12 | Glutamic acid | 0.2236 | 0.0457 | 0.0404 | 0.0260 | 0.0621 | 0.2020 |
| 13 | Phenylalanine | -0.1684 | -0.2900 | 0.2151 | -0.0948 | -0.0396 | -0.1105 |
| 14 | Putrescine | -0.1855 | 0.1238 | 0.2456 | 0.1692 | 0.2046 | 0.0311 |
| 15 | Glycerol 3-phosphate | 0.1821 | 0.0022 | 0.2151 | -0.2526 | 0.2882 | 0.0386 |
| 16 | Ornithine | -0.1611 | -0.3020 | 0.0550 | -0.1955 | -0.2537 | -0.0932 |
| 17 | Citric acid | 0.2076 | 0.0207 | 0.1648 | -0.0238 | -0.2349 | 0.1873 |
| 18 | Tetradecanoic acid | 0.1654 | -0.1191 | -0.3275 | 0.2676 | 0.0492 | -0.2927 |
| 19 | Galactose | -0.2237 | 0.0521 | 0.0465 | -0.0030 | -0.0322 | -0.2335 |
| 20 | Lysine | -0.2202 | -0.0604 | 0.0713 | -0.1106 | -0.0097 | 0.0048 |
| 21 | Tyrosine | -0.2242 | 0.0252 | 0.0460 | -0.0169 | 0.0526 | 0.0593 |
| 22 | Galactitol | -0.2220 | -0.0010 | 0.1028 | 0.0416 | -0.0230 | 0.0442 |
| 23 | Hexadecanoic acid | -0.1053 | 0.3628 | 0.0330 | -0.3656 | -0.2424 | -0.2337 |
| 24 | Inositol | -0.2222 | -0.0446 | 0.0562 | -0.0855 | -0.0393 | 0.1562 |
| 25 | 9-(E)-Octadecenoic acid | 0.2247 | -0.0240 | 0.0367 | -0.0288 | 0.0297 | 0.0239 |
| 26 | Tryptophan | -0.2231 | 0.0508 | 0.0469 | -0.0582 | 0.0313 | -0.1791 |
| 27 | Octadecanoic acid | 0.0367 | 0.4388 | -0.0983 | 0.1104 | 0.4102 | 0.0512 |
| 28 | Trehalose | 0.2047 | -0.1938 | -0.1223 | -0.0031 | -0.0441 | -0.1935 |
| 29 | Alanine | 0.2099 | 0.0831 | 0.0973 | -0.1557 | 0.1766 | 0.0166 |
